# Variation in Osteoporosis Treatment Gaps Across Fracture-Related and Elective Orthopedic Surgeries: A Single-Center Retrospective Cohort Study in Taiwan

**DOI:** 10.3390/jcm15197683

**Published:** 2026-10-04

**Authors:** Yuan-Sheng Hsu, Ya-Lian Deng, Sheng-Chieh Tseng, Cheng-Hung Lee, Kun-Hui Chen, Shih-Yi Lin

**Affiliations:** 1 Department of Orthopedic Surgery, Taichung Veterans General Hospital, Taichung 407219, Taiwan; 2 Department of Nursing, Taichung Veterans General Hospital, Taichung 407219, Taiwan; 3 Department of Post-Baccalaureate Medicine, College of Medicine, National Chung-Hsing University, Taichung 402202, Taiwan; 4 Center for Geriatrics and Gerontology, Taichung Veterans General Hospital, Taichung 407219, Taiwan; 5 School of Medicine, National Yang-Ming Chiao Tung University, Taipei 112304, Taiwan

**Keywords:** osteoporosis, secondary fracture prevention, fracture liaison service, orthopedic surgery, spinal fusion, bone mineral density

## Abstract

**Background/Objectives**: Fracture liaison services (FLSs) typically use fragility fractures as triggers to identify patients for osteoporosis care. Patients undergoing elective orthopedic surgery who already have osteoporosis documented on a preoperative densitometry report fall outside this trigger. This study compared recorded initiation of osteoporosis treatment after elective operations with that after operations performed for a fracture. A secondary, exploratory objective was to describe how preoperative T-score relates to subsequent fractures in patients without recorded treatment. **Methods**: A retrospective cohort study (2015–2025) was performed at a tertiary center in Taiwan. The source population was the institutional densitometry cohort (all patients with at least one DXA examination at the hospital), within which five index operations were identified: vertebroplasty/kyphoplasty, hip fracture internal fixation, hip arthroplasty (hemi- or total), lumbar spinal fusion, and total knee arthroplasty. Operations in patients already receiving osteoporosis therapy were excluded. The primary outcome was recorded initiation of anti-osteoporosis therapy, defined as the first dispensing at the study hospital within 365 days of surgery. Odds ratios were estimated by logistic regression with patient-clustered standard errors. **Results**: Among 6171 operations in 5739 patients, recorded one-year initiation was 28.4% overall: 46.9% after vertebroplasty, 43.1% after hip fracture fixation, 33.0% after hip arthroplasty, 17.4% after lumbar fusion, and 7.2% after total knee arthroplasty. Adjusted odds ratios versus fusion ranged from 3.16 (95% CI: 2.69–3.72) for vertebroplasty to 0.28 (0.21–0.37) for knee arthroplasty. Within hip arthroplasty, initiation was 43.9% after hemiarthroplasty, which is performed for femoral neck fracture, and 6.7% after total hip arthroplasty. Across all operations, an accompanying fracture diagnosis was associated with initiation (adjusted OR: 6.19, 5.26–7.29). The contrast persisted among the 871 operations with osteoporosis documented before surgery (48.2% after vertebroplasty, 7.5% after knee arthroplasty). In the exploratory analysis of 1971 operations without recorded treatment (99 fractures), each one-unit lower T-score was associated with a higher hazard of major osteoporotic fracture (adjusted HR: 1.25, 1.04–1.49). The estimate for the T ≤ −2.5 dichotomy was less precise (HR: 1.33, 0.83–2.12). **Conclusions**: In this selected single-center densitometry cohort, preliminary findings indicate that recorded initiation of osteoporosis treatment after orthopedic surgery followed whether the operation was performed for a fracture rather than the patient’s documented bone density, including among patients whose osteoporosis was already documented. A national reimbursement rule that requires a fracture in addition to low bone density probably contributes. Treatment outside the study hospital was not captured, so initiation proportions are lower bounds. Whether an FLS entry point keyed to existing densitometry results would close this gap remains to be tested.

## 1. Introduction

Osteoporosis is among the most consequential chronic diseases of an aging population, and hip fracture alone carries a one-year mortality of approximately 15–20% together with substantial long-term disability [1,2,3]. The first fragility fracture approximately doubles the risk of a further fracture, and that risk is front-loaded: in a national cohort of 242,108 women aged 50 years or over, the cumulative incidence of a subsequent fracture was 7.1% at one year and 12.0% at two years, with the highest rate in the first month after the index event [4,5]. Despite this, fewer than one in five patients receives appropriate treatment after the first osteoporotic fracture, a shortfall consistent enough across health systems to have acquired its own name, the osteoporosis care gap [6]. In Taiwan, fewer than one third of patients receives timely anti-osteoporotic therapy [7,8].

The consequences of a fracture in later life are not confined to the skeleton. Recovery of mobility and of independence in daily activities after a hip fracture depends heavily on cognition, frailty and the quality of post-acute care, and consensus guidance for that phase of care lists osteoporosis treatment among its core components [9]. Cognitive impairment is also a determinant of whether osteoporosis is treated at all: people with dementia sustain more fractures and fare worse after them, yet are less likely to be assessed or to receive pharmacotherapy [10,11], and agents given at long intervals by a clinician have been proposed for this group [10]. Any comparison of treatment rates between surgical populations of different age and frailty therefore has to consider cognitive comorbidity.

The Fracture Liaison Service (FLS) is a care model developed to address this issue. Its programmatic functions include case finding, patient assessment, osteoporosis assessment, treatment initiation, fall prevention, and education [12]. In a recent cohort of 4124 patients with fragility hip fractures, treatment initiation was 72.1% in an FLS setting versus 45.1% in a non-FLS setting, with better adherence [13]. National orthopedic societies have begun formal FLS accreditation programs [14], and the model has been reviewed across health systems [15].

Most of these programs are triggered by a fragility fracture. The design of case finding is based on the assumption that patients do not know they have osteoporosis and that the fracture is the first signal. In its usual form, therefore, an FLS is a secondary-prevention service: its principal entry criterion is a sentinel fracture, and although some services have added other entry routes, patients with osteoporosis who have not fractured generally fall outside its case-finding reach even when they are under the care of the same institution [12,14]. However, this does not reflect the entire patient population seen in hospitals. Specifically, it does not reflect patients undergoing lumbar spinal fusion or knee or hip arthroplasty for degenerative conditions, among whom osteoporosis is common: among patients presenting for elective arthroplasty or spinal surgery, roughly a third to two-thirds have osteoporosis on densitometry [16,17]. Further, these patients may already have undergone densitometry at the same institution showing a bone mineral density (BMD) T-score of −2.5 or below that was never acted upon. Where screening has been audited, only 18–24% of eligible patients had a preoperative dual-energy X-ray absorptiometry scan and only about 5% received pharmacotherapy [17,18]. Because a BMD evaluation is not usually made a formal part of the preoperative pathway for these operations, a fracture-triggered FLS is unlikely to enroll these patients.

The questions of whether such patients receive treatment and how their treatment compares with that of patients operated on for a fracture have not, to the authors’ knowledge, been examined directly within a single institution under one medication definition. An institutional densitometry cohort linked to operative and dispensing records was used to compare recorded initiation of anti-osteoporosis treatment across five orthopedic operations. These ranged from operations performed for a fracture (vertebroplasty and hip fracture internal fixation) through hip arthroplasty, which is performed both for femoral neck fracture and for degenerative diseases, to operations that are almost always elective (lumbar fusion and total knee arthroplasty). The primary hypothesis was directional: recorded initiation within one year would be higher after fracture-related operations than after elective operations, and this difference would persist among patients whose osteoporosis was already documented in a preoperative densitometry report. A secondary, exploratory objective was to examine how the preoperative T-score related to subsequent major osteoporotic fractures among patients who remained untreated, because the clinical importance of a treatment gap depends on the fracture risk carried by those patients.

## 2. Materials and Methods

### 2.1. Study Design and Cohort

This was a retrospective cohort study conducted at a tertiary medical center in Taiwan using a de-identified research extract covering cases from 1 January 2015 to 31 May 2025. The source population was the institutional densitometry cohort: every patient with at least one dual-energy X-ray absorptiometry (DXA) examination reported at the hospital during the study period (58,717 persons), for whom the extract links demographics, all inpatient, outpatient and emergency cases, diagnoses, procedures and dispensing at the hospital. Entry into the cohort required only that a DXA examination had been performed, for any indication and at any time relative to surgery. There was no active recruitment or sampling within the source population. All index operations recorded for cohort members during the study period were captured, but operations in patients who never underwent densitometry at the hospital were not in the extract. The cohort is therefore not a sample of all patients undergoing these operations, and the proportions reported here describe patients who were assessed by densitometry at some point. The unit of analysis was the operation, not the patient. Patients who underwent more than one class of index operation contributed to more than one group. Within-patient correlation was handled as described in Section 2.5.

### 2.2. Index Operations

Five index operations were identified from ICD-10-PCS concept names in the procedure table: vertebroplasty or kyphoplasty, internal fixation for hip fracture, hip arthroplasty, lumbar spinal fusion, and total knee arthroplasty. Hip arthroplasty comprised two implant concepts, replacement of the femoral surface (hemiarthroplasty) and replacement of the whole joint (total hip arthroplasty). They were analyzed as one group in the primary analysis, as originally specified, and separately in secondary analyses. Concept names were matched with the laterality stripped because Taiwan National Health Insurance procedure codes do not encode side, and the mapping to ICD-10-PCS defaults almost all operations to the right-sided concept. Procedure dates beyond the 31 May 2025 census were treated as sentinel values and excluded before aggregation. The index date was the operation date. For patients with repeated operations of the same class, the first was used. No age restriction was applied beyond a computable age of 30–100 years. The extract contains no information on trauma mechanisms, and external-cause codes were recorded for fewer than 1% of the operations, so a fracture could not be verified as low-trauma. Vertebroplasty and hip fracture fixation are therefore described as fracture-related operations. To document what each procedure class represents, it was determined for operations from February 2016 onward (after the transition of diagnosis coding to ICD-10-CM) whether a site-matched fracture diagnosis (ICD-10-CM S72 for hip and knee operations; S22, S32, M48.4, M48.5 or M80 for spinal operations) was recorded within 30 days before or after the index date. In a secondary analysis this accompanying fracture diagnosis was used as the exposure across all operations.

### 2.3. Exposure Ascertainment and Outcome Definition

The primary outcome was recorded initiation of pharmacotherapy for osteoporosis within 365 days of the index operation, defined as the first dispensing of bisphosphonate, denosumab, teriparatide, or raloxifene. The dispensing table covers all medications dispensed by the study hospital in inpatient, outpatient and emergency settings. It does not cover prescriptions filled at other hospitals, clinics or community pharmacies. The outcome is therefore recorded initiation at this institution, and the absence of a record is not confirmation that a patient was untreated. Agents were identified by OMOP standard concept identifier rather than by drug name, because the source field holds National Health Insurance codes rather than ingredient names. Calcium, cholecalciferol and active vitamin D analogs were not counted as initiation, whether alone or in combination. Their dispensing was tabulated separately. Initiation within 90 days is reported as a secondary timing measure. Operations in patients with any qualifying dispensing prior to the index date were excluded, so that the cohort is treatment-naïve, as far as this hospital’s records show, by design. Operations lacking at least 12 months of observable follow-up prior to the census date were excluded, so that every operation included had a full year in which initiation could occur.

One-year persistence was assessed among initiators who in addition had at least 365 days between the first dispensing and the census dates. The primary definition allowed a permissible gap of 60 days, and a stricter secondary definition a gap of 30 days, applied to the interval covered by each dispensing. For denosumab and zoledronic acid the covered interval was the labeled dosing interval (180 and 365 days, respectively). Because a single zoledronic acid infusion covers the whole observation window by construction, its persistence under this definition is not an empirical result. For that agent the results reported instead are the proportion receiving a second infusion between day 300 and day 455 among patients observable for the whole window, and an analysis restricted to short-acting agents. The specialty under which the first qualifying prescription was written and the setting in which it was issued were taken from the case associated with the dispensing.

The secondary, exploratory prognostic analysis addressed a different question from the primary analysis: not who is treated, but what fracture risk is carried by those who are not. The outcome was incident major osteoporotic fracture ascertained from ICD-10-CM codes using an inpatient-or-twice-outpatient rule. The site definition (hip, clinical spine, distal forearm and proximal humerus) follows standard descriptive-epidemiology recommendations [19]. The analysis was restricted to operations in patients aged 50 years or over with preoperative densitometry who had no recorded treatment within the first year, so that recorded pharmacotherapy could not explain differences between T-score categories. Follow-up began 180 days after surgery to blank the index event: the index fracture and its surgical aftercare continue to be coded at follow-up visits for several months, and with shorter blanking periods these re-codings cannot be separated from new fractures. The choice of 180 days is nonetheless arbitrary, so the analysis was repeated with blanking periods of 30, 90 and 365 days. Because absence of recorded treatment is defined over the first 365 days, only the 365-day blanking period is free of conditioning on events after the start of follow-up: a fracture that prompted treatment within the first year removes that operation from this analysis, which would bias the association toward the null. Follow-up ended at the first qualifying fracture or at the census date. Death recorded in the institutional data (52 deaths among the 1971 operations) is known to be incomplete, so in the main analysis it was not used as a censoring event. Sensitivity analyses censored follow-up at recorded death and at the last recorded contact with the hospital, and estimated cumulative incidence with recorded death treated as a competing event [20].

### 2.4. Densitometry, Covariates and Missing Data

Preoperative bone density was taken from the last densitometry performed at this institution on or before the day of surgery. The structured reports hold lumbar spine and femoral neck (and, for a minority, forearm) results. Total hip BMD and T-score are not present in the structured fields and could not be used. The lowest-site T-score was therefore the lower of those of the lumbar spine and the femoral neck, in keeping with the recommendation that the diagnostic classification rests on the lowest T-score of the recommended sites [21]. Patients were classified as having documented osteoporosis when that preoperative report recorded a T-score of −2.5 or below.

Two properties of the source reports require explanation. First, 74.8% of the reports (30,102 of 40,217) stated that T-scores were derived from a Caucasian female normative database without race adjustment. Every T-score analyzed here, in men as well as women, was referenced to that database. This follows IOF recommendations for standardized reporting, with the well-recognized caveats that apply when such a reference is applied to an East Asian population [22]. Second, numeric densitometry fields were stored as text, with zero used as a missing-value sentinel. T-score missingness was therefore inferred from the paired BMD field rather than from the T-score itself, since most T-scores in this cohort were legitimately negative and a positivity rule would preferentially delete osteoporotic patients. Of the 8115 operations with a computable age, 4251 (52.4%) had no densitometry before surgery and 3864 had a preoperative report. In 221 of these reports (5.7%) neither site yielded a usable T-score, leaving 3643 operations (44.9%) with a lowest-site T-score. Among the 6171 analyzed operations the corresponding figure was 2203 (35.7%). Availability was not completely at random: it differed by operation, utilization and calendar year (Table S1). No T-score was imputed. The primary analysis (Models 1 and 2) does not use the T-score and includes every analyzed operation. Analyses that require it are complete-case analyses in operations with a usable preoperative T-score, and they are interpreted as applying to patients whose bone density was measured before surgery.

**Figure 1:**
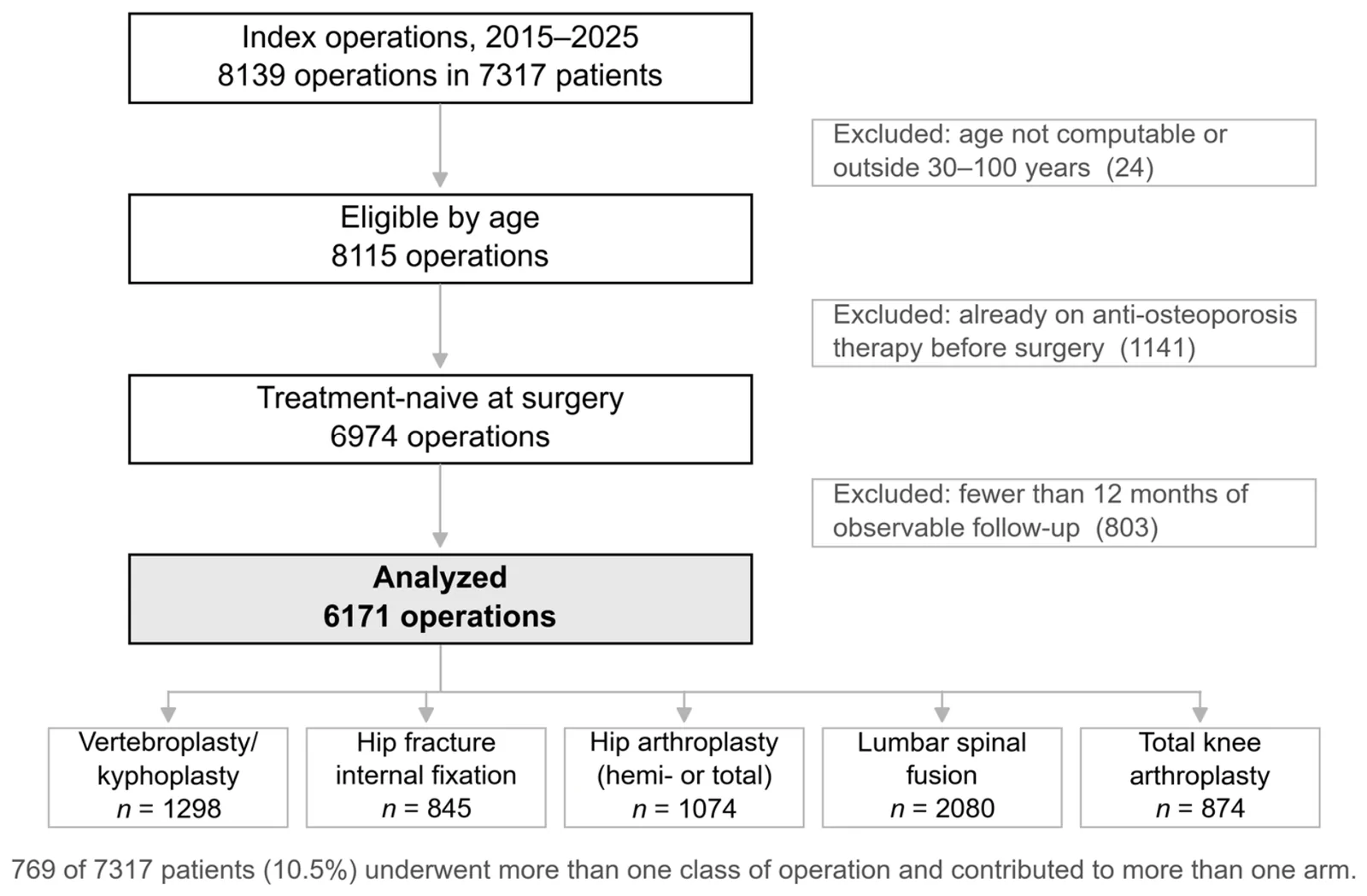
Cohort derivation. All index operations among members of the institutional densitometry cohort, 2015–2025. Of 8139 operations in 7317 patients, 24 were excluded for non-computable or out-of-range age, 1141 for anti-osteoporosis therapy already in use before surgery, and 803 for fewer than 12 months of observable follow-up, leaving 6171 operations for analysis, distributed across the five operation classes as shown. Counts are the number of operations. In total, 769 of 7317 patients (10.5%) underwent more than one class of operation and contributed to more than one arm.

Covariates were ascertained over the three years preceding each index operation: age at surgery, sex, a count of 15 predefined comorbidities (diabetes, rheumatoid arthritis, systemic lupus erythematosus, hyperthyroidism, hyperparathyroidism, chronic kidney disease, malabsorption, chronic liver disease, chronic obstructive pulmonary disease, dementia, Parkinson disease, alcohol use disorder, hypertension, coronary artery disease, and bone metastasis or myeloma; ICD-10-CM codes in Table S2), the number of outpatient visits and the number of admissions. A comorbidity was counted when at least one qualifying diagnosis code was recorded in any setting within the window. Outpatient visit count was included as a proxy for surveillance intensity in order to mitigate informed-presence bias [23]. Because it was strongly right-skewed it entered the models as log(1 + visits). Age, sex and index operation were complete after the age exclusion as shown in Figure 1. Comorbidity and utilization variables were derived from the presence of records, so the absence of a code was taken as absence of the condition and these variables had no missing values by construction. Diagnoses before 2016 were coded in ICD-9-CM and were not mapped, so comorbidity was under-ascertained for the earliest operations. A sensitivity analysis was restricted to operations from 2019 onward, for which the whole look-back window was coded in ICD-10-CM.

The regression models were descriptive and estimated whether recorded initiation differed by operation class among patients of similar age, sex, comorbidity and prior hospital use. The operation class is understood as a marker of the clinical pathway a patient enters (fracture admission versus elective admission), and the models estimate the total association of that pathway with initiation. They are not intended to isolate a causal effect of the procedure itself. A fracture that leads to the index operation is part of what defines a fracture-related pathway, so adjusting for fracture history would remove part of the contrast of interest. It was therefore not included in the primary models. Because an earlier fracture could also act as a common cause of the type of operation and of treatment, it was added in a sensitivity analysis, defined as a qualifying major osteoporotic fracture recorded more than 90 days before the index operation, together with dementia or cognitive impairment (ICD-10-CM F01–F03, G30, G31.0, G31.1, G31.83, G31.84) and recurrent oral glucocorticoid dispensing (three or more dispensing dates) in the preceding three years. Contraindications, renal function at the time of the decision, fall risk, patient refusal and clinician preference are not recorded in the extract.

### 2.5. Statistical Analysis

All analyses used Python 3.10 (statsmodels 0.14, lifelines 0.30). Baseline characteristics are median (interquartile range) or *n* (%), and proportions carry Wilson 95% confidence intervals. Recorded initiation was modeled by logistic regression in four models: Model 1 (age and sex); Model 2 (age, sex, comorbidity count, log outpatient visits, and admissions); Model 3 (Model 2 restricted to operations with osteoporosis documented before surgery); and Model 4 (Model 2 restricted to patients contributing only one class of operation), with lumbar spinal fusion as reference. Because a patient could contribute more than one operation, standard errors used a patient-clustered sandwich estimator, equivalent to generalized estimating equations with an independent working correlation [24]. Independence was preferred because the cohort is treatment-naïve by design, so the outcome of one operation depends on the patient’s other operations, a setting in which a non-diagonal working correlation can bias the estimates [25]; an exchangeable working correlation and a first-operation-only analysis are reported as sensitivity analyses. A random-intercept model was not used, as it estimates patient-specific rather than population-averaged odds ratios. Further sensitivity analyses added the DXA-to-surgery interval and were restricted to patients aged 50 or over and to operations from 2019 onward. For the prognostic analysis, Cox models adjusted for age, sex, comorbidity count and index operation with patient-clustered standard errors. The continuous T-score and the −2.5 dichotomy were fitted as separate exposures, reported side by side with Harrell’s C and the Akaike information criterion; no superiority in discrimination was tested, and dichotomizing a continuous predictor is expected to lose power [26]. To describe the shape of the T-score–fracture relationship, a four-knot restricted cubic spline of the continuous T-score was fitted in the same model (knots at the 5th, 35th, 65th and 95th percentiles: T-scores of −4.0, −2.4, −1.6 and 0.1), with a likelihood-ratio test for departure from log-linearity; a five-knot specification was not supported by 99 events. As a post hoc description, a two-piecewise Cox model [27] was fitted with its breakpoint chosen by profiling the partial likelihood over candidate T-scores from −3.5 to −1.0 in steps of 0.1, and its uncertainty assessed from the profile-likelihood interval and 500 bootstrap resamples of patients.

A causal analysis of whether treatment prevents subsequent fracture was pre-specified with an explicit falsification condition: covariate balance after inverse-probability weighting, with |SMD| < 0.1 required for every covariate. This criterion was specified before the estimates were examined. A two-sided α of 0.05 was used. The secondary and sensitivity analyses were not adjusted for multiplicity and were interpreted descriptively.

### 2.6. Reporting Guideline and Use of Generative AI

This study is reported in accordance with the Strengthening the Reporting of Observational Studies in Epidemiology (STROBE) statement for cohort studies. The completed STROBE checklist is provided as a Supplementary File.

During the preparation of this manuscript, the authors used Claude Opus 4.8 (Anthropic, San Francisco, CA, USA) for manuscript drafting. The authors have reviewed and edited all output and take full responsibility for the content of this publication.

## 3. Results

### 3.1. Cohort

In total, 8139 index operations in 7317 patients were identified. Of these patients, 769 (10.5%) underwent more than one class of operation, most often lumbar fusion with vertebroplasty (n = 197). Three groups of operations were then excluded: those with a non-computable or implausible age, those in patients already receiving anti-osteoporosis therapy, and those without 12 months of observable follow-up. This left 6171 operations in 5739 patients for analysis (Figure 1; Table S3), of whom 411 contributed more than one analyzed operation. Patients under 50 years were rare: only 145 operations were analyzed (2.3%), where 89 of them were lumbar fusions, of which only six were followed by recorded initiation.

The five groups differed substantially at baseline (Table 1). Median age ranged from 69 years (IQR: 62–76) for lumbar fusion to 80 years (72–88) for hip fracture fixation. The proportion of females ranged from 64.9% (hip fracture fixation) to 86.5% (total knee arthroplasty). Preoperative densitometry was available for 62.0% of the fusion operations and 52.9% of the vertebroplasty operations but only 19.4% of the hip fracture fixations, reflecting the timing of an emergency presentation. Because the source cohort is defined by having undergone densitometry at some point, these proportions describe the timing of densitometry relative to surgery and do not measure screening coverage. Among operations with preoperative densitometry, the prevalence of osteoporosis ranged from 35.7% (fusion) to 65.6% (vertebroplasty). In the knee arthroplasty group, 43.4% of the patients with a preoperative scan already met the osteoporosis threshold. Dementia or another cognitive impairment had been coded in the preceding three years for 6.6% of the analyzed operations, with 7.7% of vertebroplasties, 11.8% of hip fracture fixations and 12.0% of hip arthroplasties, against 2.7% of fusions and 2.6% of knee arthroplasties (Table S4).

Healthcare utilization did not parallel the treatment gradient described below: fusion patients had the fewest prior outpatient visits (median of eight over three years, IQR: 4–28) and knee arthroplasty patients had the most (median of 20, IQR: 7–48).

Diagnosis codes showed what the procedure classes represented (Table S5). Among the operations from February 2016 onward, a site-matched fracture diagnosis was recorded within 30 days of 97.9% of the vertebroplasties and 99.9% of the hip fracture fixations. The same code also accompanied 72.6% of the hip arthroplasties, 23.7% of the lumbar fusions and 1.8% of the knee arthroplasties. The hip arthroplasty group was itself mixed. Of its 1074 analyzed operations, 759 (70.7%) were hemiarthroplasties, the standard operation for a displaced femoral neck fracture (median age of 79 years; hip fracture diagnosis in 99.6%). The remaining 315 were total hip arthroplasties (median age of 68 years; hip fracture diagnosis in 8.5%). A neoplastic or pathological-fracture code accompanied 3.9% of the vertebroplasties, and whether a fracture was low-trauma could not be established for any group.

### 3.2. Recorded Treatment Initiation by Index Operation

**Figure 2:**
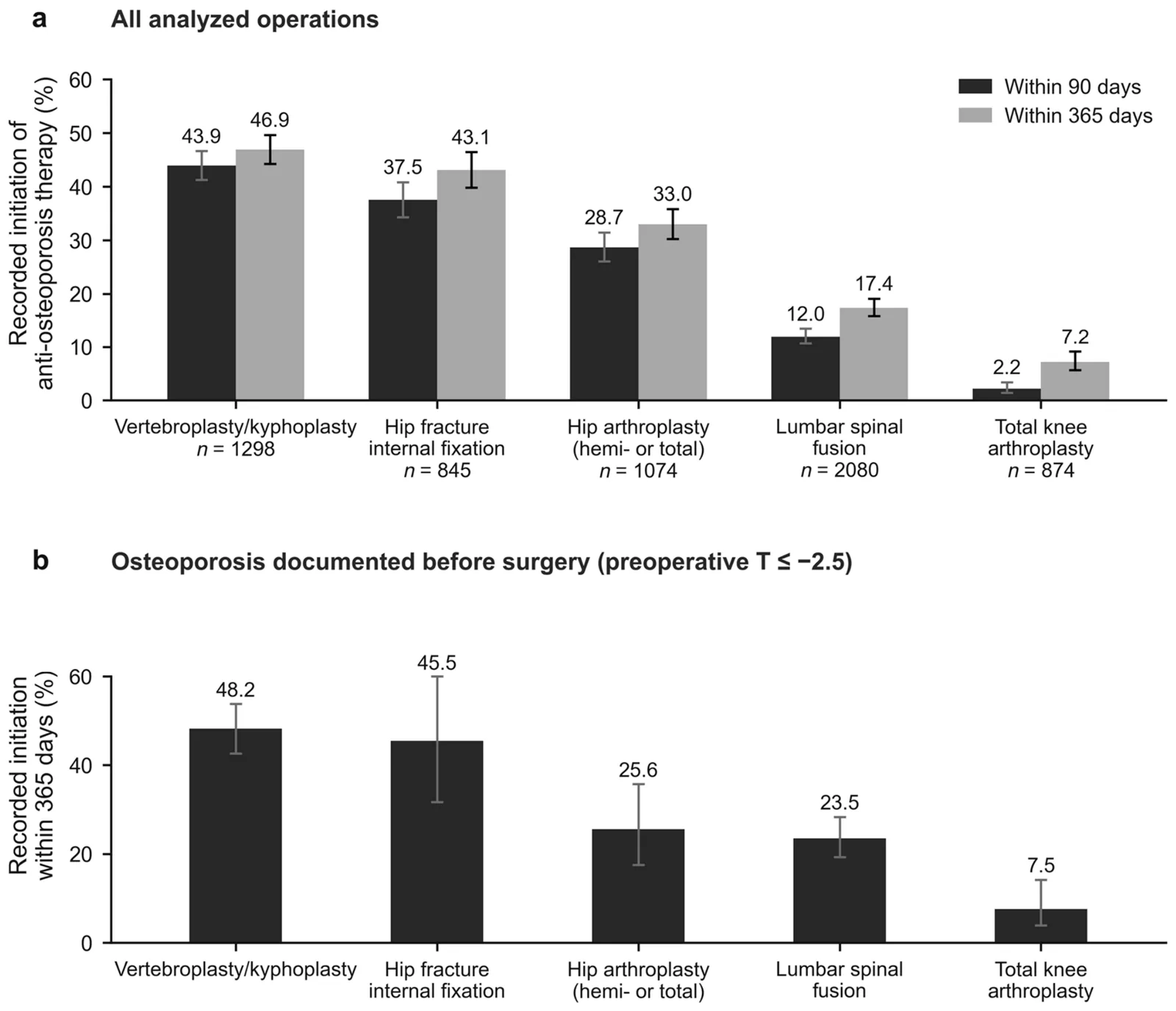
Recorded initiation of anti-osteoporosis treatment after orthopedic surgery. (**a**) Proportion of all analyzed operations with recorded initiation within 90 days and within 365 days, by operation, ordered by the 365-day proportion. (**b**) Proportion with recorded initiation within 365 days restricted to the 871 operations with osteoporosis documented before surgery (preoperative T ≤ −2.5). Error bars are Wilson 95% confidence intervals. Hip arthroplasty includes hemiarthroplasty (70.7% of the group) and total hip arthroplasty. Numerators and denominators are in Table 2. Adjusted odds ratios are in Table 3.

Recorded one-year initiation differed more than six-fold across the five operations. It was 46.9% (95% CI: 44.2–49.6) after vertebroplasty, 43.1% (39.8–46.4) after hip fracture fixation, 33.0% (30.2–35.8) after hip arthroplasty, 17.4% (15.8–19.1) after lumbar spinal fusion and 7.2% (5.7–9.1) after total knee arthroplasty (Table 2, Figure 2a). Overall, 1752 of the 6171 operations (28.4%) were followed by recorded initiation within one year, and the same ordering held at 90 days (43.9% down to 2.2%). Calcium or an active vitamin D analog, which did not count as initiation, had been dispensed within one year after 261 of the 4419 operations without recorded initiation (5.9%; Table S6).

Adjusting for age and sex (Model 1) resulted in odds ratios versus fusion of 3.15 for vertebroplasty, 2.61 for hip fracture fixation, 1.84 for hip arthroplasty and 0.30 for knee arthroplasty (Table 3). Adding comorbidity count and healthcare utilization (Model 2) changed these little, with 3.16 (2.69–3.72), 2.57 (2.12–3.13), 1.82 (1.53–2.17) and 0.28 (0.21–0.37). These covariates were themselves associated with initiation (comorbidity count of 1.09 per condition; outpatient visits of 1.18 per unit of log[1 + visits]), so they did not explain the differences between operations. The gradient was robust to within-patient clustering, to restriction to single-operation patients (Model 4) and to the first operation of each patient, and to an exchangeable working correlation (Table S7).

The intermediate position of hip arthroplasty reflected its composition. Recorded initiation was 43.9% (40.4–47.4) after hemiarthroplasty and 6.7% (4.4–10.0) after total hip arthroplasty (Table 2). When analyzed separately, hemiarthroplasty showed a treatment pattern similar to hip fracture fixation, whereas total hip arthroplasty showed a pattern similar to knee arthroplasty. The adjusted odds ratios versus fusion were 2.81 (2.31–3.41) for hemiarthroplasty, indistinguishable from hip fracture fixation (2.73, 2.24–3.32), and 0.33 (0.21–0.53) for total hip arthroplasty, close to knee arthroplasty (0.28, 0.21–0.38). Lumbar fusion was heterogeneous in the same way: recorded initiation was 36.5% (32.1–41.0) when a vertebral or osteoporotic fracture diagnosis accompanied the fusion and 12.0% (10.4–13.7) when it did not. The pattern held across all operations. Taking an accompanying site-matched fracture diagnosis as the exposure (from February 2016 onward), recorded initiation was 43.7% (1362/3115) with such a diagnosis and 9.8% (243/2487) without it (adjusted odds ratio: 6.19, 5.26–7.29; Table S5).

The estimates were stable in further sensitivity analyses (Table S8): restriction to patients aged 50 years or over (3.15, 2.54, 1.84 and 0.28), and restriction to operations from 2019 onward (2.97, 2.61, 1.71 and 0.23). Patients with coded dementia or cognitive impairment were not less likely to have recorded initiation (48.5% versus 27.0% without; adjusted odds ratio: 1.28, 0.98–1.65), the crude difference reflecting their concentration in the fracture-related operations.

### 3.3. The Treatment Gradient Persists Among Patients with Documented Osteoporosis

Osteoporosis prevalence differed markedly across the five operations, so the gradient could in principle reflect differing pre-test probability; restriction to patients with documented osteoporosis argued against that. Among the 871 operations (in 829 patients) whose own preoperative densitometry already recorded a T-score of −2.5 or below, recorded initiation within one year was 48.2% (146/303; 95% CI: 42.6–53.8) after vertebroplasty, 45.5% (20/44; 31.7–59.9) after hip fracture fixation, 25.6% (22/86; 17.5–35.7) after hip arthroplasty, 23.5% (78/332; 19.3–28.3) after lumbar fusion and 7.5% (8/106; 3.9–14.2) after knee arthroplasty (Table 2, Figure 2b). Adjusted odds ratios versus fusion were 3.11 (2.18–4.42), 2.29 (1.15–4.57), 1.01 (0.57–1.77) and 0.22 (0.10–0.49) (Table 3, Model 3). The hip fracture fixation estimate was imprecise (44 operations, 20 initiations) and the subgroup was not powered for it, so the contrast rested mainly on vertebroplasty and knee arthroplasty. The subgroup was doubly selected, by densitometry and by its result, and that selection differed by operation (a usable preoperative T-score in 18.0% of the hip fracture fixations up to 57.8% of the fusions). The gradient persisted after adjustment for the DXA-to-surgery interval and in first-operation and exchangeable-correlation analyses (Tables S7 and S8).

Two features of Model 3 deserve mention. First, the pooled hip arthroplasty group was not distinguishable from lumbar fusion (1.01, 0.57–1.77), but it was mixed: its 86 operations comprised 55 hemiarthroplasties (18 initiations, 32.7%) and 31 total hip arthroplasties (four initiations, 12.9%). Entered separately, their odds ratios were 1.42 (0.75–2.69) and 0.44 (0.14–1.35), too imprecise to separate. The fracture theme held again: with an accompanying fracture diagnosis as the exposure, recorded initiation in this subgroup was 42.7% (210/492) with such a diagnosis and 14.7% (49/333) without it (adjusted odds ratio: 4.51, 3.09–6.56; Table S5). The pattern is therefore better described as a contrast between fracture-accompanied and other operations than as a five-step gradient. Second, age and sex were not associated with recorded initiation in this subgroup (1.00 per year, 0.98–1.02; female: 1.00, 0.66–1.51), whereas both were in the whole cohort. Among patients with documented densitometric osteoporosis then, operation class remained associated with recorded initiation after adjustment for the measured covariates. This does not establish that the operation itself is responsible for the remaining variation.

Among knee arthroplasty patients with documented osteoporosis, 98 of 106 (92.5%) had no recorded initiation. Knee arthroplasty had been expected to act as a partial negative control, on the assumption that these patients had higher bone density. That assumption was not borne out: a total of 43.4% of those scanned before surgery had densitometric osteoporosis. Whether non-treatment was appropriate for individual patients cannot be judged from the available variables.

Within lumbar fusion alone, initiation tracked the preoperative densitometry category as expected (23.5% if osteoporotic, 12.0% if osteopenic, 2.2% if normal; age- and sex-adjusted odds ratio for documented osteoporosis: 2.34, 1.61–3.40, *p* = 8.7 × 10^−6^; Table S9, Figure S1). Within an operation class, the densitometry result was thus associated with prescribing.

### 3.4. Persistence and Prescriber

Of the 1752 operations followed by recorded initiation, 1709 had at least 365 days between the first dispensing and the census dates and were eligible for the persistence analysis; the remaining 43 were excluded. One-year persistence with a 60-day permissible gap was 80.5% overall and varied little by operation (77.5% to 82.4%), so the between-operation differences arose almost entirely at initiation rather than at continuation (Table S10, Figure S2). Persistence differed far more by agent than by operation: under the stricter 30-day definition it was 81.6% for six-monthly denosumab, 47.3% for oral bisphosphonates and 43.1% for raloxifene. Yearly zoledronic acid is not comparable under this definition, because a single infusion covers the whole window by construction. Among 97 zoledronic acid initiators observable long enough, 53 (54.6%, 95% CI: 44.7–64.2) received a second infusion between day 300 and day 455. Counting any anti-osteoporosis agent in that window, 58 (59.8%) did.

Orthopedics wrote 66.2% of the first prescriptions and neurosurgery wrote 24.1% (Table S11, Figure S3). The split was near-even for the two spinal operations (fusion was 50.8% for orthopedics versus 46.4% for neurosurgery), whereas hip fracture fixation was concentrated in orthopedics (76.6%) with a further 16.5% from a combined geriatric–orthopedic service. Only 7.9% of the first prescriptions were written during an inpatient stay. Median time from surgery to first prescription rose from 14 days after vertebroplasty to 123 days after knee arthroplasty.

### 3.5. Exploratory Analysis: Preoperative T-Score and Subsequent Fracture in Patients Without Recorded Treatment

**Figure 3:**
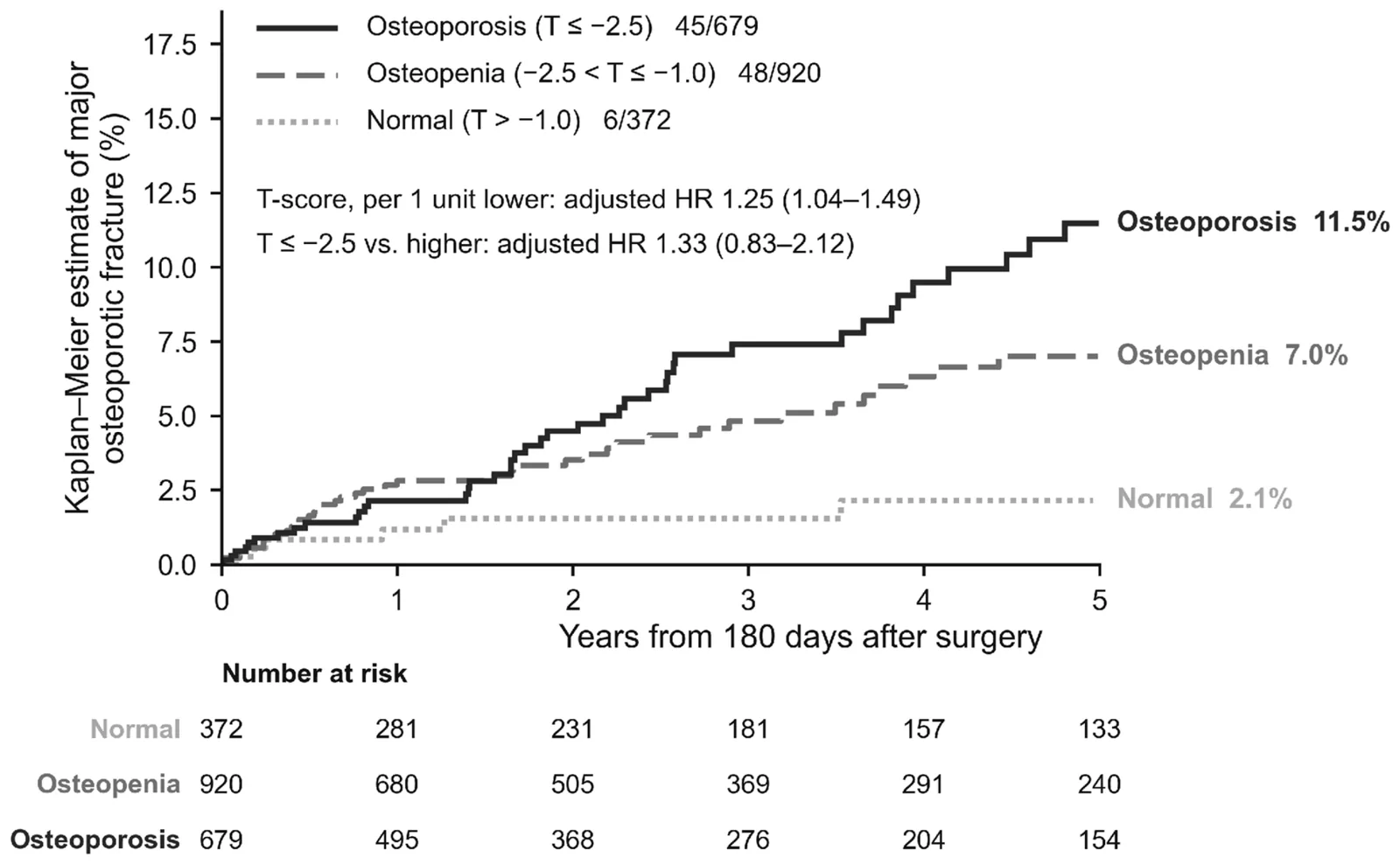
Major osteoporotic fracture by preoperative densitometry category (exploratory analysis). Each curve is one minus the Kaplan–Meier estimate, that is, the estimated percentage of operations followed by a major osteoporotic fracture by a given time, with follow-up beginning 180 days after surgery to blank the index event, restricted to the 1971 operations in patients aged 50 or over with preoperative densitometry and no recorded treatment in the first year. Categories are defined by the lower of lumbar spine and femoral neck T-scores. The legend gives fractures/operations for each category (osteoporosis: 45/679, osteopenia: 48/920, normal: 6/372), and the percentages at the right are the estimates at five years (11.5%, 7.0% and 2.1%). Numbers still under observation at each year are shown beneath the *x*-axis. Death was not treated as a competing event in this figure because its recording is incomplete. Estimates treating recorded death as a competing event were identical to one decimal place (Table S14). The two hazard ratios printed in the panel are from separate Cox models adjusted for age, sex, comorbidity count and index operation, with patient-clustered standard errors.

Among 1971 operations (1881 patients) in patients aged 50 or over with preoperative densitometry and no recorded treatment in the first year, 99 major osteoporotic fractures occurred (Table 4, Figure 3). Incidence rose with worsening bone status: it was 4.3 per 1000 person–years (95% CI: 1.6–9.5) when the preoperative scan was normal, 16.1 (11.8–21.3) when osteopenic and 21.7 (15.8–29.0) when osteoporotic. The normal-category rate rests on only six events, so the rate ratio for osteopenia versus normal (3.7, 95% CI: 1.6–8.6) is imprecise, and the osteopenic and osteoporotic intervals overlap. Each one-unit lower T-score was associated with an adjusted hazard ratio of 1.25 (1.04–1.49; *p* = 0.015). The estimate for the T ≤ −2.5 dichotomy was weaker and less precise, at 1.33 (0.83–2.12; *p* = 0.24). Its confidence interval includes the null but is also compatible with a clinically relevant association, and the two specifications differ little in discrimination (Harrell’s C of 0.686 versus 0.678, difference of 0.008, bootstrap 95% CI: −0.010 to 0.021; AIC of 1351.0 versus 1355.2; Table S12). These data therefore do not show that the threshold lacks prognostic value.

The continuous estimate was stable when the follow-up start was varied from 30 to 365 days (Table S13). Censoring at recorded death or at the last hospital contact did not change it, and five-year cumulative incidence estimated with recorded death as a competing event matched the Kaplan–Meier estimate to one decimal place, reflecting incomplete death ascertainment rather than unimportant competing mortality (Table S14). Only the 365-day landmark avoids conditioning on treatment status after follow-up began.

A restricted cubic spline gave only borderline evidence of departure from log-linearity (*p* = 0.052), with hazard rising across the osteopenic range and flattening below a T-score of about −2.5; a post hoc two-piecewise model could not identify a stable breakpoint (Tables S15–S17, Figure S4). No conclusion about a threshold is therefore drawn.

## 4. Discussion

In the 6171 orthopedic operations among the members of a single-center densitometry cohort, recorded initiation of anti-osteoporosis therapy within one year varied more than six-fold across five operations, and the ordering followed whether the operation was performed for a fracture. The association persisted when the analysis was restricted to the 871 operations in patients whose own preoperative densitometry already documented osteoporosis, a group in whom densitometric osteoporosis, though not necessarily suitable for treatment, was on record at the same hospital before the operation. Two further observations point the same way: within hip arthroplasty and within lumbar fusion, the operations accompanied by a fracture diagnosis were followed by recorded initiation three to seven times as often as those that were not.

The possible implication for FLS design is specific. The care gap described in the FLS literature is largely framed as a failure of case finding: patients are not identified, so they are not treated [6,12]. In the documented-osteoporosis subgroup described here, identification in the densitometric sense had already occurred: the examination had been performed, at the same hospital, before the operation, and the report recorded a T-score in the osteoporotic range. What was not observed is the step from that report to a prescription, and its frequency was associated with the type of admission. An FLS whose inclusion criterion is a fracture event would be unlikely to enroll most of the 332 lumbar fusion patients or 106 knee arthroplasty patients with documented osteoporosis described here through the index admission, although 23 (6.9%) and 20 (18.9%) of them, respectively, had an earlier major osteoporotic fracture on record and could have entered a service by that route. An entry criterion keyed to the existence of a densitometry report in the osteoporotic range (which the hospital already held) would in principle reach them, although in Taiwan it would also need reimbursement criteria that cover patients without a fracture. This study did not evaluate such a pathway. Its feasibility, effectiveness, cost-effectiveness and effect on fractures are questions for a prospective evaluation.

The prescriber data suggested where such a service could be integrated into clinical care. Two specialties wrote nearly all the first prescriptions, and for spinal operations they split almost evenly between orthopedics and neurosurgery. Only 7.9% of the prescriptions were written before discharge. A pathway in which responsibility is shared between two surgical services and discharged in the outpatient setting weeks to months later is the kind of configuration a coordinator role is designed to address. The persistence data indicate that the between-operation difference lies at initiation rather than continuation: once started, 77.5–82.4% of the patients persisted regardless of operation, and the largest differences were between agents rather than between operations.

Several alternative explanations warrant consideration. The first is reimbursement. For most of the study period Taiwan’s National Health Insurance reimbursed anti-osteoporosis medication principally for patients with both densitometric low bone mass and a vertebral or hip fracture [8]. A patient with a T-score of −2.5 or below and no such fracture was therefore generally ineligible for reimbursed treatment, whatever the surgeon intended. This is probably the most important structural explanation of the contrast: recorded initiation tracked an accompanying fracture diagnosis (43.7% versus 9.8%) more closely than the operation class. It does not account for everything, since 57% of apparently eligible patients (documented osteoporosis plus a fracture diagnosis) still have no recorded initiation, and a prior major fracture is associated with lower, not higher, odds of initiation in that subgroup (0.44, 0.26–0.76). The second is deliberate deferral: spine surgeons may withhold antiresorptive therapy after instrumented fusion out of concern for bone healing, although best-practice guidance recommends treating rather than withholding [28]. Deferral would predict delayed rather than absent treatment, yet 1718 of 2080 fusion operations (82.6%) had no recorded qualifying prescription within a full year, and where one occurred the median interval was 40 days. The third is transfer of responsibility, which bears directly on the primary outcome: patients discharged to rehabilitation, another hospital or primary care may be treated there, and post-discharge pathways plausibly differ by operation, so misclassification may be differential. Fracture-related operations more often follow an emergency admission and discharge to rehabilitation or long-term care, whereas elective operations follow a scheduled pathway with outpatient follow-up at the same hospital. If anything, transfer should be more common after hip fracture than after elective arthroplasty, biasing the comparison toward the null, but this cannot be verified here. As a bound, for unrecorded treatment alone to explain the contrast, about 30% of all fusion patients (the gap between 46.9% and 17.4%) would have had to start treatment elsewhere while no untreated vertebroplasty patient did; among observed prescriptions, the two surgical specialties wrote 90.3%. The fourth is that the elective groups were healthier and needed treatment less; the association was nevertheless present within the documented-osteoporosis subgroup and unchanged by adjustment for comorbidity, prior utilization, cognitive impairment and glucocorticoid dispensing. The fifth is residual confounding by unmeasured factors, including contraindications, renal function, fracture severity, fall risk, patient refusal and clinician preference. A T-score of −2.5 or below documents densitometric osteoporosis; it does not establish that every such patient has an immediate indication for, or would have accepted, pharmacotherapy. Finally, selection into the densitometry cohort differed by operation: patients who reach densitometry are those in whom osteoporosis was already suspected, which probably raises treatment proportions in every group and need not do so equally. Operations without a preoperative T-score were followed by recorded initiation more often than those with one (33.1% versus 19.9%), probably because patients first scanned after surgery entered through a densitometry that reimbursement ties to treatment. This selection differed by operation, but Model 2 refitted within the 2203 operations with a preoperative T-score gave the same pattern (3.86, 3.20, 0.98 and 0.23; Table S8).

The exploratory prognostic analysis addresses a separate question and carries less inferential weight, resting as it does on 99 events. Among patients with no recorded treatment, a lower preoperative T-score was associated with a higher hazard of subsequent major osteoporotic fracture, and this association was stable across blanking periods and censoring rules. The estimate for the −2.5 dichotomy was in the same direction and less precise. It excluded the null only with the shortest blanking period. This is not interpreted as evidence that the threshold fails to predict fracture: dichotomization discards information and power [26]; discrimination was similar for the two specifications, and no formal comparison of predictive performance was undertaken. Fracture incidence in the osteopenic category (16.1 per 1000 person–years) was higher than in the normal category, although the latter rested on six events. This is consistent with the well-established observation that a large share of fragility fractures occurs in people whose T-score lies above the osteoporosis threshold [29,30], even though screening and treatment criteria remain keyed to that line [31]. The apparent flattening of risk below a T-score of about −2 to −2.5 was of borderline significance; its location could not be identified, and no biological or clinical interpretation is attached to it. Throughout, these are statements of association in patients without recorded treatment. They are neither validated predictions nor estimates of what treatment would achieve.

The continuous association is consistent with the established gradient of risk, in which fracture risk rises by a factor of about 1.5 for each standard deviation decrease in bone mineral density, without a step at any particular value [32]. A diagnostic cut-off such as −2.5 serves classification and reimbursement, but it assigns the same status to patients whose risks differ widely, and a different status to patients on either side of the line whose risks are nearly equal. Contemporary risk stratification follows the continuous model. FRAX combines femoral neck bone mineral density, entered as a continuous variable, with age, sex and clinical risk factors to give a ten-year fracture probability [33], and recent guidelines add a very-high-risk category defined by features such as a recent fracture, a very low T-score or a high fracture probability [34]. FRAX could not be calculated here, because height, weight and several clinical risk factors were not available, so this study cannot show how the patients without recorded treatment would be classified under such strategies. The present observation is narrower: among patients without recorded treatment, fracture risk varied across the whole range of the T-score, which was compatible with stratification on a continuous scale.

Coded dementia or cognitive impairment was not associated with lower recorded initiation in this cohort (adjusted odds ratio: 1.28, 0.98–1.65), whereas a Japanese nationwide study of 474,293 patients with hip fracture found lower use of anti-osteoporotic medication among those with cognitive impairment [11], in keeping with the wider literature [10]. Diagnosis codes under-ascertain cognitive impairment; entry to a densitometry cohort is selective, and in these data such patients are concentrated in the fracture-related operations, so the difference is not interpreted. Frailty, functional status and activities of daily living were not measured.

The setting matters for generalization. The reimbursement rule discussed above is specific to Taiwan’s single-payer National Health Insurance and would not apply in systems that reimburse primary prevention. Taiwan also has one of the denser FLS networks in the Asia-Pacific region, organized, as elsewhere, around the sentinel fracture [35,36]. Other features may also differ between cultures and health systems, although no data on them were available: the role of family members in medication decisions for older adults, patients’ freedom to consult any hospital without referral, which dispenses prescribing across institutions, and the acceptability of long-term pharmacotherapy. The direction of the finding, under-treatment of osteoporosis before and after elective arthroplasty and spinal surgery, has been reported from North American and South Asian centers [16,17,18], which suggests that it is not peculiar to one culture. The magnitude of the contrast should not be assumed to transfer.

Several limitations should be addressed. First and most importantly, the primary outcome captures only dispensing at this institution. Patients who obtained medication in the community or at another hospital are misclassified as untreated, so all absolute non-treatment proportions are upper bounds, and the misclassification may differ by operation. The data are not linked to National Health Insurance claims, so the proportion treated elsewhere cannot be quantified. Second, the cohort is drawn from a densitometry registry at a tertiary referral center; it is not representative of all patients undergoing these operations; selection into it differed by operation (preoperative densitometry in 19.4% to 62.0%), and the reported proportions should not be read as institution-wide treatment rates. These preliminary findings apply to this selected single-center cohort and require confirmation in broader multicenter populations that include patients with and without DXA examinations. Third, the unit of analysis is the operation, and 10.5% of patients appear in more than one group; standard errors were clustered by patient, and single-operation (Model 4) and first-operation analyses gave nearly identical estimates. Fourth, death ascertainment is incomplete: only institutionally recorded deaths are captured, about one fifth (0.221) of those expected, and capture declines with age, partly because terminally ill patients in Taiwan are often discharged home to die. Death is a competing event for fracture. In the prognostic cohort a death before any fracture was recorded for 51 of the 1971 operations (2.6%); if capture here resembled the whole cohort the true proportion loss would be roughly 12%. As an outer bound, censoring at the last recorded hospital contact removed 36% of the person–time (6446 to 4131 person–years) and moved the hazard ratio per unit lower T-score from 1.25 to 1.29 (Table S14). Because unrecorded death is likelier in older patients with lower T-scores, the resulting downward bias in incidence and Kaplan–Meier estimates is expected to be shifted toward the null for the T-score association, but cannot be quantified without the national death registry, so the prognostic results should be regarded as exploratory. Fifth, indication cannot be fully observed: some untreated patients may have had contraindications, and the data record no patient refusal, planned deferral or referral to primary care. Sixth, height, weight and BMI were unavailable, precluding BMI adjustment and any BMD-free clinical FRAX comparison. Seventh, all T-scores derive from a Caucasian female normative database applied to a Taiwanese population. Relative to an ethnicity- and sex-specific reference, this lowers T-scores in East Asian women, classifying more as osteoporotic, and raises them in men, classifying fewer [22]. The documented-osteoporosis subgroup (85% female) therefore probably includes some who would be osteopenic under a local reference and omits some men; this affects who enters the subgroup, does not differ by operation, and is the classification on which clinicians and reimbursement rules actually acted. Eighth, the operation groupings rest on ICD-10-PCS concept-name matching, reviewed by the Department of Orthopedic Surgery; the hip arthroplasty class pools hemiarthroplasty and total hip arthroplasty; fracture mechanism is not recorded, and diagnoses before 2016 are coded in ICD-9-CM and not mapped, so residual misclassification cannot be excluded. Ninth, the proposed case-finding criterion is itself constrained by access to densitometry: it presupposes an existing report, and which patients are scanned is governed by capacity, scheduling and reimbursement, so patients who never reach a scan remain outside a densitometry-keyed entry point just as they fall outside a fracture-keyed one. Finally, this study describes an association between the type of operation and recorded treatment; it does not show that treating these patients would prevent fractures. The attempt to estimate that effect failed its pre-specified balance criterion: after inverse-probability weighting, age (standardized mean difference: 0.17) and preoperative T-score (−0.31) remained imbalanced (Tables S18 and S19), so treated and untreated patients differed in measured prognostic factors that weighting could not reconcile, and must be presumed to differ in unmeasured ones such as contraindications, frailty, refusal and treatment obtained elsewhere. Under those conditions no adjustment available in these data can yield a causal estimate of treatment efficacy, and none is offered.

This study also has strengths. Five operations were compared within one institution under a single medication definition and a single dispensing source, so differences in data capture between hospitals did not enter the comparison. Operative, densitometry and dispensing records were linked at the patient level, which allowed the analysis to be restricted to patients whose osteoporosis was documented before surgery. The cohort was large (6171 operations), and the main contrast was robust to patient-level clustering, to restriction to the first operation of each patient and to a range of sensitivity analyses (Tables S7 and S8). The T-score was analyzed on a continuous scale as well as at the conventional threshold.

The findings have possible practical implications for surgeons, preoperative clinics and FLS coordinators. First, a densitometry report in the osteoporotic range that already exists at the time of elective surgery is an opportunity for assessment that requires no new test, and preoperative assessment could check for it routinely. Second, services whose entry criterion is a fracture could consider whether patients with such a report can also be referred. Third, only 7.9% of the first prescriptions were written before discharge and two surgical specialties wrote 90.3% of them, so responsibility for osteoporosis follow-up after discharge needs to be assigned explicitly. For policy, reimbursement criteria that require a fracture limit what any such pathway can achieve in Taiwan. These implications are derived from observational associations and are not tested interventions.

Future research should first test prospectively whether an entry point keyed to existing densitometry results increases the rate of appropriate treatment and reduces fractures, ideally with linkage to national claims and death registries so that treatment and death outside the hospital are captured. A second possible direction follows from the accessibility constraint: opportunistic screening from imaging that surgical patients already undergo. Deep-learning models can estimate bone mineral density or flag osteoporosis from chest radiographs [37,38] and from routine CT [39], and best-practice guidelines for elective spinal reconstruction already endorse opportunistic CT assessment where formal densitometry has not been obtained [28]. 

The impact of imaging-derived flags on treatment at the point of surgery was not examined. Additional imaging information may also inform the evaluation of older orthopedic patients. The letter by Syrmos et al. describes applications of nuclear medicine in evaluating periprosthetic infection, prosthetic loosening and other skeletal disorders [40]. These applications concern specific diagnostic questions; the article does not establish a role for these modalities in routine osteoporosis screening or in closing treatment gaps. Nuclear medicine imaging was not evaluated in the present study.

## 5. Conclusions

In this densitometry cohort, initiation of anti-osteoporosis therapy after orthopedic surgery tracked whether the operation was performed for, or accompanied by, a fracture, rather than the patient’s documented bone density. A reimbursement rule that requires a fracture in addition to low bone density probably contributes. These preliminary findings describe associations in a selected single-center cohort that did not capture treatment obtained elsewhere, and the exploratory fracture analysis is not a validated prediction rule. A case-finding criterion based on an existing diagnostic densitometry result, rather than on a fracture event, is a directly testable extension of the FLS model.

## Figures and Tables

**Table 1:** Baseline characteristics by index operation. The 8115 operations have a computable age of 30–100 years; the last row provides the 6171 cases analyzed for treatment initiation (Figure 1).

Characteristic	All Operations	Vertebroplasty/Kyphoplasty	Hip Fracture Internal Fixation	Hip Arthroplasty (Hemi- or Total)	Lumbar Spinal Fusion	Total Knee Arthroplasty
Operations, n	8115	1937	1021	1315	2690	1152
Age at surgery, median (IQR), years	74 (67–81)	77 (70–83)	80 (72–88)	77 (69–84)	69 (62–76)	72 (67–78)
Female sex, n (%)	6066 (74.8)	1496 (77.2)	663 (64.9)	921 (70.0)	1990 (74.0)	996 (86.5)
Preoperative DXA available, n (%)	3864 (47.6)	1025 (52.9)	198 (19.4)	391 (29.7)	1669 (62.0)	581 (50.4)
Interval DXA-to-surgery, median, years	0.2	0.1	1.9	1.2	0.1	1.5
Lowest-site T-score, median (IQR)	−2.4 (−3.1–−1.6)	−2.8 (−3.4–−2.1)	−3.0 (−3.5–−2.1)	−2.5 (−3.2–−1.7)	−2.0 (−2.7–−1.2)	−2.2 (−2.9–−1.4)
Osteoporosis (T ≤ −2.5) among those with a densitometric T-score, n (%)	1751 (48.1)	639 (65.6)	120 (65.2)	193 (52.3)	556 (35.7)	243 (43.4)
Comorbidity count (0–15), median (IQR)	1 (0–2)	1 (0–2)	1 (0–2)	1 (0–2)	1 (0–2)	1 (0–2)
Outpatient visits in prior 3 y, median (IQR)	13 (4–38)	15 (4–43)	17 (2–42)	15 (4–42)	8 (4–28)	20 (7–48)
Already on anti-osteoporosis therapy before surgery, n (%)	1141 (14.1)	416 (21.5)	115 (11.3)	153 (11.6)	260 (9.7)	197 (17.1)
Analyzed for treatment initiation, n	6171	1298	845	1074	2080	874

Hip arthroplasty comprises hemiarthroplasty (replacement of the femoral surface; 759 of the 1074 analyzed operations) and total hip arthroplasty (315). DXA, dual-energy X-ray absorptiometry; IQR, interquartile range.

**Table 2:** Recorded initiation of anti-osteoporosis treatment. Initiation is the first dispensing at the study hospital. The 95% confidence intervals are Wilson intervals.

Operation	All Analyzed, n	Initiated Within 90 Days, n (%)	Initiated Within 365 Days, n (%; 95% CI)	No Recorded Initiation Within 365 Days, n (%)	Documented Osteoporosis, n	Of These, Initiated Within 365 Days, n (%; 95% CI)	Of These, No Recorded Initiation, n (%)
Vertebroplasty/kyphoplasty	1298	570 (43.9)	609 (46.9; 44.2–49.6)	689 (53.1)	303	146 (48.2; 42.6–53.8)	157 (51.8)
Hip fracture internal fixation	845	317 (37.5)	364 (43.1; 39.8–46.4)	481 (56.9)	44	20 (45.5; 31.7–59.9)	24 (54.5)
Hip arthroplasty (hemi- or total)	1074	308 (28.7)	354 (33.0; 30.2–35.8)	720 (67.0)	86	22 (25.6; 17.5–35.7)	64 (74.4)
Hip arthroplasty of which hemiarthroplasty	759	300 (39.5)	333 (43.9; 40.4–47.4)	426 (56.1)	55	18 (32.7; 21.8–45.9)	37 (67.3)
Hip arthroplasty of which total hip arthroplasty	315	8 (2.5)	21 (6.7; 4.4–10.0)	294 (93.3)	31	4 (12.9; 5.1–28.9)	27 (87.1)
Lumbar spinal fusion	2080	250 (12.0)	362 (17.4; 15.8–19.1)	1718 (82.6)	332	78 (23.5; 19.3–28.3)	254 (76.5)
Total knee arthroplasty	874	19 (2.2)	63 (7.2; 5.7–9.1)	811 (92.8)	106	8 (7.5; 3.9–14.2)	98 (92.5)
All operations	6171	1464 (23.7)	1752 (28.4; 27.3–29.5)	4419 (71.6)	871	274 (31.5; 28.5–34.6)	597 (68.5)

**Table 3:** Adjusted odds of recorded initiation of anti-osteoporosis therapy within 365 days. Odds ratios (95% CI) from logistic regression with patient-clustered standard errors. Lumbar spinal fusion is the reference category.

Term	Model 1: Age + Sex	Model 2: Model 1 + Comorbidity and Utilization	Model 3: Model 2, Documented Osteoporosis Only	Model 4: Model 2, Single-Operation Patients Only
Lumbar spinal fusion	1.00 (reference)	1.00 (reference)	1.00 (reference)	1.00 (reference)
Vertebroplasty/kyphoplasty	3.15 (2.68–3.71)	3.16 (2.69–3.72)	3.11 (2.18–4.42)	3.33 (2.77–4.01)
Hip fracture internal fixation	2.61 (2.15–3.17)	2.57 (2.12–3.13)	2.29 (1.15–4.57)	2.61 (2.11–3.24)
Hip arthroplasty (hemi- or total)	1.84 (1.54–2.19)	1.82 (1.53–2.17)	1.01 (0.57–1.77)	1.96 (1.61–2.38)
Total knee arthroplasty	0.30 (0.23–0.40)	0.28 (0.21–0.37)	0.22 (0.10–0.49)	0.27 (0.19–0.38)
Age, per year	1.04 (1.04–1.05)	1.04 (1.04–1.05)	1.00 (0.98–1.02)	1.04 (1.04–1.05)
Female sex (vs. male)	1.84 (1.60–2.12)	1.96 (1.69–2.27)	1.00 (0.66–1.51)	1.94 (1.66–2.26)
Comorbidity count (0–15), per condition	–	1.09 (1.03–1.15)	1.16 (1.00–1.35)	1.09 (1.02–1.16)
Outpatient visits in prior 3 y, per unit of log(1 + visits)	–	1.18 (1.11–1.26)	1.27 (1.06–1.51)	1.17 (1.09–1.25)
Admissions in prior 3 y, per admission	–	0.89 (0.84–0.94)	0.86 (0.76–0.98)	0.91 (0.86–0.96)
Operations/patients/initiations	6171/5739/1752	6171/5739/1752	871/829/274	5106/5106/1462

Model 3 is restricted to operations with a preoperative lowest-site T-score ≤ −2.5, and Model 4 to patients who contributed a single class of index operation. Alternative treatments of within-patient clustering are in Table S7. Single-class status was defined among all 8139 identified operations, before the exclusions in Figure 1. Hemiarthroplasty and total hip arthroplasty are entered separately in Table S5.

**Table 4:** Preoperative densitometry and subsequent major osteoporotic fracture (exploratory analysis). Hazard ratios are adjusted for age, sex, comorbidity count and index operation, with patient-clustered standard errors. The normal category contains six fractures, so rate ratios relative to it are imprecise.

Preoperative DXA Category	Operations, n	Fractures, n	Person–Years	Incidence, per 1000 Person–Years (95% CI)	Rate Ratio vs. Normal (95% CI)
Normal (T > −1.0)	372	6	1379	4.3 (1.6–9.5)	1.0 (reference)
Osteopenia (−2.5 < T ≤ −1.0)	920	48	2991	16.1 (11.8–21.3)	3.7 (1.6–8.6)
Osteoporosis (T ≤ −2.5)	679	45	2076	21.7 (15.8–29.0)	5.0 (2.1–11.7)
T-score, per 1-unit lower—adjusted HR (95% CI)	1971	99		1.248 (1.043–1.493)	*p* = 0.015
T-score ≤ −2.5 vs. higher—adjusted HR (95% CI)	1971	99		1.325 (0.827–2.122)	*p* = 0.24

## Data Availability

The aggregate data supporting the conclusions of this article are included within the article and its Supplementary File. The underlying patient-level records cannot be shared publicly because they are held under the institutional data governance of Taichung Veterans General Hospital; requests may be directed to the corresponding authors and are subject to approval by Institutional Review Board.

## Supplementary Materials

The following supporting information can be downloaded at https://www.mdpi.com/article/10.3390/jcm15197683/s1, Table S1: Availability of a preoperative T-score and its predictors; Table S2: The 15 comorbidities and their ICD-10-CM ascertainment codes; Table S3: Cohort derivation counts by operation; Table S4: Cognitive impairment, prior fracture and glucocorticoid dispensing by operation; Table S5: Fracture diagnoses accompanying each index operation, and initiation within hip arthroplasty and lumbar fusion by fracture status; Table S6: Calcium and vitamin D analogue dispensing; Table S7: Initiation models under alternative treatments of within-patient clustering; Table S8: Sensitivity analyses of the initiation models; Table S9: Treatment initiation after lumbar spinal fusion by preoperative densitometry category; Table S10: One-year persistence among those who started treatment; Table S11: Specialty writing the first anti-osteoporosis prescription; Table S12: Continuous versus dichotomized T-score: fit and discrimination; Table S13: Prognostic analysis with alternative blanking periods; Table S14: Prognostic analysis under alternative censoring rules and with death as a competing event; Table S15: Adjusted hazard ratios at fixed T-scores from the restricted cubic spline; Table S16: Comparison of linear, spline, and two-piecewise models for the T-score–fracture relationship; Table S17: Profile likelihood and bootstrap stability of the two-piecewise breakpoint; Table S18: Treated versus untreated: why these data cannot support a causal estimate; Table S19: Covariate balance before and after inverse-probability weighting; Figure S1: Initiation after lumbar fusion by preoperative densitometry category; Figure S2: The osteoporosis care cascade after orthopedic surgery. For each operation: all treatment-naïve operations (grey), those initiating within one year (blue), and those still on treatment at one year (green). Almost the entire loss occurs at the first step; Figure S3: Which specialty writes the first anti-osteoporosis prescription. Departments contributing at least 1% are shown separately; the remainder are pooled; Figure S4: Dose–response of preoperative T-score and major osteoporotic fracture. The completed STROBE checklist for cohort studies is provided as a separate Supplementary File.

## Abbreviations

AIC	Akaike information criterion
BMD	bone mineral density
CI	confidence interval
DXA	dual-energy X-ray absorptiometry
FLS	fracture liaison service
HR	hazard ratio
ICD-10-CM	International Classification of Diseases, Tenth Revision, Clinical Modification
ICD-10-PCS	International Classification of Diseases, Tenth Revision, Procedure Coding System
IPTW	inverse-probability-of-treatment weighting
IQR	interquartile range
MOF	major osteoporotic fracture
OR	odds ratio
STROBE	Strengthening the Reporting of Observational Studies in Epidemiology
SMD	standardized mean difference
OMOP	Observational Medical Outcomes Partnership
IOF	International Osteoporosis Foundation

## References

[1] Compston J.E., McClung M.R., Leslie W.D. (2019). Osteoporosis. Lancet.

[2] Kanis J.A., Norton N., Harvey N.C., Jacobson T., Johansson H., Lorentzon M., McCloskey E.V., Willers C., Borgström F. (2021). SCOPE 2021: A new scorecard for osteoporosis in Europe. Arch. Osteoporos..

[3] Brauer C.A., Coca-Perraillon M., Cutler D.M., Rosen A.B. (2009). Incidence and mortality of hip fractures in the United States. JAMA.

[4] Banefelt J., Åkesson K.E., Spångeus A., Ljunggren O., Karlsson L., Ström O., Ortsäter G., Libanati C., Toth E. (2019). Risk of imminent fracture following a previous fracture in a Swedish database study. Osteoporos. Int..

[5] Bliuc D., Nguyen N.D., Milch V.E., Nguyen T.V., Eisman J.A., Center J.R. (2009). Mortality risk associated with low-trauma osteoporotic fracture and subsequent fracture in men and women. JAMA.

[6] Wang M., Seibel M.J. (2024). Secondary fracture prevention in primary care: A narrative review. Osteoporos. Int..

[7] Lee M.-T., Fu S.-H., Hsu C.-C., Chen H.-M., Lin J.-W., Tsai K.-S., Hwang J.-S., Lin S.-C., Wu C.-H., Wang C.-Y. (2023). Epidemiology and clinical impact of osteoporosis in Taiwan: A 12-year trend of a nationwide population-based study. J. Formos. Med. Assoc..

[8] Wang C.-Y., Fu S.-H., Hung C.-C., Yang R.-S., Lin J.-W., Chen H.-M., Hsiao F.-Y., Shen L.-J. (2022). Impact of the requirement of bone mineral density evidence on utilization of anti-osteoporosis medications in patients with hip fracture in Taiwan. Int. J. Health Policy Manag..

[9] Reyes B.J., Mendelson D.A., Mujahid N., Mears S.C., Gleason L., Mangione K.K., Nana A., Mijares M., Ouslander J.G. (2020). Postacute management of older adults suffering an osteoporotic hip fracture: A consensus statement from the International Geriatric Fracture Society. Geriatr. Orthop. Surg. Rehabil..

[10] Shrestha P., Basida B., Elam R.E., El Hajj J., Le B., Fink H.A., Bethel M., Tharrington S.G., Carbone L.D. (2025). Disparities in the diagnosis and treatment of osteoporosis in persons with cognitive impairment and dementia. Curr. Osteoporos. Rep..

[11] Mori Y., Tarasawa K., Tanaka H., Mori N., Fushimi K., Aizawa T., Fujimori K. (2025). Increased risk of postoperative complications in elderly hip fracture patients with cognitive impairment: Evidence from a Japanese nationwide database. Geriatr. Gerontol. Int..

[12] Kates S.L., Miclau T. (2022). The fracture liaison service: Its history, current state, how it works, and future directions. OTA Int..

[13] Mazza O., Gluck C., Menkes-Caspi N., Bornstein R.J., Amir H., Bahar M., Haim A. (2025). Inpatient rehabilitation fracture liaison service (FLS) improves outcomes for secondary prevention of hip fractures. Bone Rep..

[14] Tarantino U., Greggi C., Visconti V.V., Cariati I., Bonanni R., Gasperini B., Iundusi R., Gasbarra E., Leali P.T., Brandi M.L. (2023). Fracture liaison service model: Project design and accreditation. Osteoporos. Int..

[15] Lim W.X., Khor H.M., Lee J.K., Ong T. (2023). Fracture liaison service and its role in secondary fracture prevention in Malaysia: A scoping review. Malays. Orthop. J..

[16] Dave D., Bhattacharjee S.K., Shah D.D., Mascerhans A., Dey P.C., Arumugan S., Mehra V., Agarwal V., Garg S., Gore S.C. (2022). Osteoporosis in Indian patients undergoing elective arthroplasty and spinal procedures: An observational study. Cureus.

[17] Bernatz J.T., Brooks A.E., Squire M.W., Illgen R.I., Binkley N.C., Anderson P.A. (2019). Osteoporosis is common and undertreated prior to total joint arthroplasty. J. Arthroplasty.

[18] Shepard S., Bartholomew A., Houserman D., Bamberger H.B., Manocchio A.G. (2024). Assessing osteoporosis screening compliance in total joint surgery: A retrospective chart review. J. Osteopath. Med..

[19] Kanis J.A., Adachi J.D., Cooper C., Clark P., Cummings S.R., Diaz-Curiel M., Harvey N., Hiligsmann M., Papaioannou A., Pierroz D.D. (2013). Standardizing the descriptive epidemiology of osteoporosis: Recommendations from the Epidemiology and Quality of Life Working Group of IOF. Osteoporos. Int..

[20] Austin P.C., Lee D.S., Fine J.P. (2016). Introduction to the analysis of survival data in the presence of competing risks. Circulation.

[21] Shuhart C.R., Yeap S.S., Anderson P.A., Jankowski L.G., Lewiecki E.M., Morse L.R., Rosen H.N., Weber D.R., Zemel B.S., Shepherd J.A. (2019). Executive summary of the 2019 ISCD Position Development Conference on monitoring treatment, DXA cross-calibration and least significant change, spinal cord injury, peri-prosthetic and orthopedic bone health, transgender medicine, and pediatrics. J. Clin. Densitom..

[22] Wáng Y.X.J., Blake G.M., Xiao B.-H., Guglielmi G., Su Y., Jiang Y., Guermazi A., Kwok T.C.Y., Griffith J.F. (2024). East Asians’ T-scores for the diagnosis of osteoporosis should be calculated using ethnicity- and gender-specific BMD reference ranges: Justifications. Skelet. Radiol..

[23] Goldstein B.A., Bhavsar N.A., Phelan M., Pencina M.J. (2016). Controlling for informed presence bias due to the number of health encounters in an electronic health record. Am. J. Epidemiol..

[24] Zeger S.L., Liang K.Y. (1986). Longitudinal data analysis for discrete and continuous outcomes. Biometrics.

[25] Pepe M.S., Anderson G.L. (1994). A cautionary note on inference for marginal regression models with longitudinal data and general correlated response data. Commun. Stat. Simul. Comput..

[26] Royston P., Altman D.G., Sauerbrei W. (2006). Dichotomizing continuous predictors in multiple regression: A bad idea. Stat. Med..

[27] Muggeo V.M.R. (2003). Estimating regression models with unknown break-points. Stat. Med..

[28] Sardar Z.M., Coury J.R., Cerpa M.M., DeWald C.J., Ames C.P., Shuhart C.M., Watkins C., Polly D.W., Dirschl D.R., Klineberg E.O. (2022). Best Practice Guidelines for Assessment and Management of Osteoporosis in Adult Patients Undergoing Elective Spinal Reconstruction. Spine.

[29] Siris E.S., Brenneman S.K., Miller P.D., Barrett-Connor E., Chen Y.-T., Sherwood L.M., Abbott T.A. (2004). Predictive value of low BMD for 1-year fracture outcomes is similar for postmenopausal women ages 50-64 and 65 and older: Results from NORA. J. Bone Miner. Res..

[30] Wainwright S.A., Marshall L.M., Ensrud K.E., Cauley J.A., Black D.M., Hillier T.A., Hochberg M.C., Vogt M.T., Orwoll E.S. (2005). Hip fracture in women without osteoporosis. J. Clin. Endocrinol. Metab..

[31] Curry S.J., Krist A.H., Owens D.K., Barry M.J., Caughey A.B., Davidson K.W., Doubeni C.A., Epling J.W., Kemper A.R., US Preventive Services Task Force (2018). Screening for osteoporosis to prevent fractures: US Preventive Services Task Force recommendation statement. JAMA.

[32] Marshall D., Johnell O., Wedel H. (1996). Meta-analysis of how well measures of bone mineral density predict occurrence of osteoporotic fractures. BMJ.

[33] Kanis J.A., Harvey N.C., Johansson H., Liu E., Vandenput L., Lorentzon M., Leslie W.D., McCloskey E.V. (2020). A decade of FRAX: How has it changed the management of osteoporosis?. Aging Clin. Exp. Res..

[34] Camacho P.M., Petak S.M., Binkley N., Diab D.L., Eldeiry L.S., Farooki A., Harris S.T., Hurley D.L., Kelly J., Lewiecki E.M. (2020). American Association of Clinical Endocrinologists/American College of Endocrinology clinical practice guidelines for the diagnosis and treatment of postmenopausal osteoporosis—2020 update. Endocr. Pract..

[35] Chang L.-Y., Tsai K.-S., Peng J.-K., Chen C.-H., Lin G.-T., Lin C.-H., Tu S.-T., Mao I.-C., Gau Y.-L., Liu H.-C. (2018). The development of Taiwan Fracture Liaison Service network. Osteoporos. Sarcopenia.

[36] Chan D.-C., Chang L.-Y., Akesson K.E., Mitchell P., Chen C.-H., Lewiecki E.M., Lee J.K., Lau T.C., Songpatanasilp T., Lee K.B. (2018). Consensus on best practice standards for Fracture Liaison Service in the Asia-Pacific region. Arch. Osteoporos..

[37] Jang M., Kim M., Bae S.J., Lee S.H., Koh J.-M., Kim N. (2022). Opportunistic Osteoporosis Screening Using Chest Radiographs with Deep Learning: Development and External Validation with a Cohort Dataset. J. Bone Miner. Res..

[38] Asamoto T., Takegami Y., Sato Y., Takahara S., Yamamoto N., Inagaki N., Maki S., Saito M., Imagama S. (2024). External validation of a deep learning model for predicting bone mineral density on chest radiographs. Arch. Osteoporos..

[39] Westerhoff M., Gyftopoulos S., Dane B., Vega E., Murdock D., Lindow N., Herter F., Bousabarah K., Recht M.P., Bredella M.A. (2025). Deep Learning-based Opportunistic CT Osteoporosis Screening and the Establishment of Normative Values. Radiology.

[40] Syrmos C.H.N., Lytras D., Giannouli V. (2017). The Remarkable Applications of Nuclear Medicine in Orthopedic. Sci. J. Nucl. Med. Radiat. Ther..

