# Supplementary material for: Variation in Osteoporosis Treatment Gaps Across Fracture-Related and Elective Orthopedic Surgeries: A Single-Center Retrospective Cohort Study in Taiwan
Source: J Clin Med. 2026 Oct 4;15(19):7683. doi: 10.3390/jcm15197683 (PMC13666248; doi:10.3390/jcm15197683)
Supplement: Supplementary file 1 [file jcm-15-07683-s001.zip › STROBE.pdf]

STROBE Statement — Checklist of items for cohort studies

Variation in Osteoporosis Treatment Gaps Across Fracture-Related and Elective Orthopedic Surgeries: A Single-Center Retrospective Cohort Study in Taiwan

Item	No.	Recommendation	Reported on page/section
Title and abstract	1	(a) Indicate the study's design with a commonly used term in the title or the abstract	Title (“A Single-Center Retrospective Cohort Study in Taiwan”); Abstract
		(b) Provide in the abstract an informative and balanced summary of what was done and what was found	Abstract
**Introduction**			
Background/rationale	2	Explain the scientific background and rationale for the investigation being reported	Introduction, ¶1–2
Objectives	3	State specific objectives, including any prespecified hypotheses	Introduction, last ¶ (directional primary hypothesis; secondary exploratory objective)
**Methods**			
Study design	4	Present key elements of study design early in the paper	Materials and Methods, Study design and cohort
Setting	5	Describe the setting, locations, and relevant dates, including periods of recruitment, exposure, follow-up, and data collection	Materials and Methods, Study design and cohort
Participants	6	(a) Give the eligibility criteria, and the sources and methods of selection of participants. Describe methods of follow-up	Section 2.1 (densitometry cohort as source population; no sampling); Section 2.2
		(b) For matched studies, give matching criteria and number of exposed and unexposed	N/A (unmatched cohort)
Variables	7	Clearly define all outcomes, exposures, predictors, potential confounders, and effect modifiers. Give diagnostic criteria, if applicable	Sections 2.2–2.4 (recorded initiation; fracture-related operations; covariates listed)
Data sources/measurement	8	For each variable of interest, give sources of data and details of methods of assessment (measurement). Describe comparability of assessment methods if there is more than one group	Sections 2.3–2.4 (coverage of the dispensing table; comorbidity codes in Table S2)
Bias	9	Describe any efforts to address potential sources of bias	Section 2.4 (estimand; informed-presence adjustment); Section 2.5 (clustering; sensitivity analyses); Section 4
Study size	10	Explain how the study size was arrived at	Results, Cohort; Fig. 1
Quantitative variables	11	Explain how quantitative variables were handled in the analyses. If applicable, describe which groupings were chosen and why	Section 2.4 (log[1 + visits]; lowest-site T-score); Section 2.5 (spline knots)

Statistical methods	12	(a) Describe all statistical methods, including those used to control for confounding	Section 2.5 (patient-clustered standard errors; Cox models)
		(b) Describe any methods used to examine subgroups and interactions	Section 2.5 (Models 3–4; hip arthroplasty and fusion by fracture diagnosis)
		(c) Explain how missing data were addressed	Section 2.4, ¶2–3 (rates, mechanism, complete-case strategy); Table S1
		(d) If applicable, explain how loss to follow-up was addressed	Section 2.3 (censoring rules; death as competing event); Table S14; Section 4, Limitations (fourth item)
		(e) Describe any sensitivity analyses	Section 2.5; Table S7, Table S8, Table S13, Table S14, Table S17
**Results**			
Participants	13	(a) Report numbers of individuals at each stage of study — e.g. numbers potentially eligible, examined for eligibility, confirmed eligible, included in the study, completing follow-up, and analysed	Section 3.1; Figure 1; Table S3
		(b) Give reasons for non-participation at each stage	Results, Cohort; Fig. 1
		(c) Consider use of a flow diagram	Fig. 1
Descriptive data	14	(a) Give characteristics of study participants and information on exposures and potential confounders	Table 1
		(b) Indicate number of participants with missing data for each variable of interest	Section 2.4; Table 1; Table S1
		(c) Summarise follow-up time	Table 1
Outcome data	15	Report numbers of outcome events or summary measures over time	Tables 2 and 4; Figures 2–3
Main results	16	(a) Give unadjusted estimates and, if applicable, confounder-adjusted estimates and their precision	Table 2 (unadjusted, with 95% CIs); Table 3 (Models 1–4); Table 4
		(b) Report category boundaries when continuous variables were categorized	Table 4 (T-score categories); Section 2.4
		(c) If relevant, consider translating estimates into meaningful clinical measures	Table 4 (incidence per 1,000 person-years); Figure 3
Other analyses	17	Report other analyses done — e.g. analyses of subgroups and interactions, and sensitivity analyses	Sections 3.2, 3.3 and 3.5; Table S5, Table S7, Table S8, Table S12–Table S17
**Discussion**			
Key results	18	Summarise key results with reference to study objectives	Discussion, ¶1
Limitations	19	Discuss limitations of the study, taking into account sources of potential bias or imprecision	Section 4, alternative explanations and Limitations (ten items, with direction of bias)
Interpretation	20	Give a cautious overall interpretation of results considering objectives, limitations, multiplicity of analyses, results from similar studies, and other relevant evidence	Section 4, ¶1–3 and prognostic ¶; Section 5

Generalisability	21	Discuss the generalisability (external validity) of the study results	Section 4, ¶ "The setting matters for generalization"; Limitations (second item)
**Other information**			
Funding	22	Give the source of funding and the role of the funders for the present study and, if applicable, for the original study on which the present article is based	Funding
