# Supplementary material for: Variation in Osteoporosis Treatment Gaps Across Fracture-Related and Elective Orthopedic Surgeries: A Single-Center Retrospective Cohort Study in Taiwan
Source: J Clin Med. 2026 Oct 4;15(19):7683. doi: 10.3390/jcm15197683 (PMC13666248; doi:10.3390/jcm15197683)
Supplement: Supplementary file 1 [file jcm-15-07683-s001.zip › Supplementary_Files.pdf]

Supplementary Materials

Variation in Osteoporosis Treatment Gaps Across Fracture-Related and Elective Orthopedic Surgeries: A Single-Center Retrospective Cohort Study in Taiwan

Yuan-Sheng Hsu, Ya-Lian Deng, Sheng-Chieh Tseng, Cheng-Hung Lee, Kun-Hui Chen, Shih-Yi Lin

Table S1. Availability of a preoperative T-score and its predictors

(a) Availability among the 8,115 operations with a computable age

	**Vertebroplasty/kyphoplasty**	**Hip fracture internal fixation**	**Hip arthroplasty (hemi- or total)**	**Lumbar spinal fusion**	**Total knee arthroplasty**	**All operations**
Operations, n	1,937	1,021	1,315	2,690	1,152	8,115
No preoperative DXA report, n (%)	912 (47.1)	823 (80.6)	924 (70.3)	1,021 (38.0)	571 (49.6)	4,251 (52.4)
Preoperative report, n	1,025	198	391	1,669	581	3,864
Report without a usable lowest-site T-score, n (% of reports)	51 (5.0)	14 (7.1)	22 (5.6)	113 (6.8)	21 (3.6)	221 (5.7)
Spine T-score unusable, n (% of reports)	142 (13.9)	30 (15.2)	57 (14.6)	231 (13.8)	75 (12.9)	535 (13.8)
Femoral neck T-score unusable, n (% of reports)	156 (15.2)	48 (24.2)	89 (22.8)	266 (15.9)	112 (19.3)	671 (17.4)
Usable lowest-site T-score, n (% of operations)	974 (50.3)	184 (18.0)	369 (28.1)	1,556 (57.8)	560 (48.6)	3,643 (44.9)

(b) Predictors of having no usable preoperative T-score among the 6,171 analyzed operations (logistic regression, patient-clustered standard errors)

**Term**	**OR for having no usable preoperative T-score (95% CI)**	**p**
Vertebroplasty/kyphoplasty (vs. lumbar fusion)	1.93 (1.65–2.25)	<0.001
Hip fracture internal fixation (vs. lumbar fusion)	10.83 (8.38–14.00)	<0.001
Hip arthroplasty (hemi- or total) (vs. lumbar fusion)	5.03 (4.18–6.06)	<0.001
Total knee arthroplasty (vs. lumbar fusion)	2.14 (1.82–2.52)	<0.001
Age, per year	1.00 (0.99–1.01)	0.747
Female sex	0.78 (0.68–0.89)	<0.001
Comorbidity count, per condition	1.09 (1.03–1.15)	0.003
Outpatient visits, per unit of log(1 + visits)	0.69 (0.65–0.73)	<0.001
Admissions, per admission	1.02 (0.98–1.06)	0.269
Calendar year of surgery, per year	0.90 (0.88–0.92)	<0.001

(c) Recorded initiation within 365 days by T-score availability

Operation	With T-score: initiated n/N (%)	Without T-score: initiated n/N (%)
Vertebroplasty/kyphoplasty	211/503 (41.9)	398/795 (50.1)
Hip fracture internal fixation	33/83 (39.8)	331/762 (43.4)
Hip arthroplasty (hemi- or total)	33/203 (16.3)	321/871 (36.9)
Lumbar spinal fusion	146/1,090 (13.4)	216/990 (21.8)
Total knee arthroplasty	16/324 (4.9)	47/550 (8.5)
All operations	439/2,203 (19.9)	1,313/3,968 (33.1)

A T-score was treated as unusable when the paired BMD field was missing, zero or outside 0.3–2.0 g/cm², or the T-score lay outside ±8. Joint Wald test of all predictors in (b), i.e. of missingness completely at random with respect to the observed covariates: $\chi^2 = 804.5$, 10 df, $p < 0.001$. Availability therefore depends on observed characteristics, chiefly the operation (emergency fracture surgery precedes densitometry), prior contact with the hospital and calendar year. Whether it also depends on the unobserved T-score itself cannot be tested. T-scores were not imputed; analyses requiring a T-score are complete-case analyses.

Table S2. The 15 comorbidities and their ICD-10-CM ascertainment codes

Condition	ICD-10-CM code prefixes
Diabetes mellitus	E10, E11
Rheumatoid arthritis	M05, M06
Systemic lupus erythematosus	M32
Hyperthyroidism	E05
Hyperparathyroidism	E21
Chronic kidney disease	N18
Malabsorption	K90
Chronic liver disease	K70, K74
Chronic obstructive pulmonary disease	J44
Dementia	F01, F03
Parkinson disease	G20
Alcohol use disorder	F10
Hypertension	I10
Coronary artery disease	I25
Bone metastasis or multiple myeloma	C79.5, C90.0

A condition was counted when at least one diagnosis with a listed prefix was recorded in any setting (inpatient, outpatient or emergency) in the three years before the index operation. The comorbidity count is the number of conditions present (0–15). Diagnoses before 2016 were coded in ICD-9-CM and were not mapped.

Table S3. Cohort derivation counts by operation

Operation	Underwent operation	Age computable and 30-100 years	Not on therapy before surgery	At least 12 months observable follow-up (analyzed)
Vertebroplasty/kyphoplasty	1,939	1,937	1,521	1,298
Hip fracture internal fixation	1,030	1,021	906	845
Hip arthroplasty (hemi- or total)	1,323	1,315	1,162	1,074
Lumbar spinal fusion	2,695	2,690	2,430	2,080
Total knee arthroplasty	1,152	1,152	955	874

The per-operation version of Figure 1.

Table S4. Cognitive impairment, prior fracture and glucocorticoid dispensing by operation

	Vertebroplasty/kyphoplasty	Hip fracture internal fixation	Hip arthroplasty (hemi- or total)	Lumbar spinal fusion	Total knee arthroplasty	All operations
Analyzed, n	1,298	845	1,074	2,080	874	6,171

Dementia/cognitive impairment in prior 3 y, n (%)	100 (7.7)	100 (11.8)	129 (12.0)	56 (2.7)	23 (2.6)	408 (6.6)
Prior MOF (>90 d before surgery), n (%)	72 (5.5)	42 (5.0)	64 (6.0)	58 (2.8)	48 (5.5)	284 (4.6)
Recurrent oral glucocorticoid (≥3 dispensing dates in prior 3 y), n (%)	70 (5.4)	41 (4.9)	60 (5.6)	109 (5.2)	63 (7.2)	343 (5.6)
Documented osteoporosis: prior MOF, n/N (%)	33/303 (10.9)	8/44 (18.2)	25/86 (29.1)	23/332 (6.9)	20/106 (18.9)	109/871 (12.5)
Initiated, cognitive impairment n/N (%)	59/100 (59.0)	49/100 (49.0)	61/129 (47.3)	25/56 (44.6)	4/23 (17.4)	198/408 (48.5)
Initiated, no cognitive impairment (%)	45.9	42.3	31.0	16.7	6.9	27.0

Analyzed operations (n = 6,171). Dementia/cognitive impairment: ICD-10-CM F01–F03, G30, G31.0, G31.1, G31.83 or G31.84 in the three years before surgery. Prior MOF: a qualifying major osteoporotic fracture recorded more than 90 days before the index operation. Glucocorticoid: three or more dispensing dates of oral prednisolone or methylprednisolone in the three years before surgery (dispensing end dates are largely missing, so cumulative days could not be computed).

Table S5. Fracture diagnoses accompanying each index operation, and initiation within hip arthroplasty and lumbar fusion by fracture status

(a) Diagnoses recorded within 30 days before or after the index operation (analyzed operations from February 2016, after the transition to ICD-10-CM)

	Vertebroplasty/kyphoplasty	Hip fracture internal fixation	Hip arthroplasty (hemi- or total)	Lumbar spinal fusion	Total knee arthroplasty
Operations from Feb 2016 (ICD-10-CM era), n	1,218	766	959	1,884	775
Site-matched fracture diagnosis within ±30 d, n (%)	1,193 (97.9)	765 (99.9)	696 (72.6)	447 (23.7)	14 (1.8)
Neoplastic/pathological code within ±30 d, n (%)	48 (3.9)	14 (1.8)	9 (0.9)	36 (1.9)	<5 (0.4)
External-cause (V/W) code within ±30 d, n (%)	<5 (0.1)	<5 (0.5)	<5 (0.2)	<5 (0.1)	<5 (0.1)

(b) Hip arthroplasty by implant concept (all 1,074 analyzed operations)

	Hemiarthroplasty (femoral surface)	Total hip arthroplasty
Analyzed, n	759	315
Age, median	79	68
Initiated within 365 d, n (%; 95% CI)	333 (43.9; 40.4–47.4)	21 (6.7; 4.4–10.0)
Hip-fracture diagnosis within ±30 d (operations from Feb 2016), n/N (%)	672/675 (99.6)	24/284 (8.5)

(c) Hip arthroplasty by accompanying hip fracture diagnosis (operations from February 2016)

Hip arthroplasty (hemi- or total)	n	Initiated within 365 d, n	% (95% CI)	Age, median
with a hip-fracture diagnosis within ±30 d	696	298	42.8 (39.2–46.5)	79.0
without	263	17	6.5 (4.1–10.1)	68.0

(d) Lumbar spinal fusion by accompanying vertebral or osteoporotic fracture diagnosis (operations from February 2016)

Lumbar spinal fusion	n	Initiated within 365 d, n	% (95% CI)	Age, median
with a vertebral/osteoporotic fracture diagnosis within ±30 d	447	163	36.5 (32.1–41.0)	74
without	1,437	172	12.0 (10.4–13.7)	68

(e) Model 2 with hip arthroplasty split by fracture diagnosis (operations from February 2016; reference lumbar spinal fusion)

Term	OR vs lumbar fusion (95% CI)
Hip fracture internal fixation	2.63 (2.14–3.23)
Hip arthroplasty without a hip fracture diagnosis	0.32 (0.19–0.53)
Hip arthroplasty with a hip fracture diagnosis	2.60 (2.12–3.19)
Total knee arthroplasty	0.25 (0.18–0.34)
Vertebroplasty/kyphoplasty	3.23 (2.72–3.83)

(f) Hemiarthroplasty and total hip arthroplasty: recorded initiation, all analyzed operations and the documented-osteoporosis subgroup

Operation	All analyzed, n	Initiated within 90 d, n/N (%; 95% CI)	Initiated within 365 d, n/N (%; 95% CI)	Documented osteoporosis: initiated within 365 d, n/N (%; 95% CI)
Hemiarthroplasty	759	300/759 (39.5; 36.1–43.0)	333/759 (43.9; 40.4–47.4)	18/55 (32.7; 21.8–45.9)
Total hip arthroplasty	315	8/315 (2.5; 1.3–4.9)	21/315 (6.7; 4.4–10.0)	4/31 (12.9; 5.1–28.9)

(g) Models 2 and 3 with hemiarthroplasty and total hip arthroplasty entered separately (all analyzed operations; odds ratios versus lumbar spinal fusion)

	Model 2, six groups	Model 3, six groups (documented osteoporosis)
n	6,171	871
events	1,752	274
Hemiarthroplasty	2.81 (2.31–3.41)	1.42 (0.75–2.69)
Hip fracture internal fixation	2.73 (2.24–3.32)	2.31 (1.16–4.62)
Total knee arthroplasty	0.28 (0.21–0.38)	0.22 (0.10–0.48)
Total hip arthroplasty	0.33 (0.21–0.53)	0.44 (0.14–1.35)
Vertebroplasty/kyphoplasty	3.28 (2.79–3.86)	3.13 (2.20–4.45)

(h) An accompanying site-matched fracture diagnosis as the exposure, across all operations from February 2016 (Model 2 covariates; operation class not in the model)

	All analyzed operations from Feb 2016	Documented osteoporosis
n	5,602	825
With a site-matched fracture diagnosis: initiated n/N (%)	1,362/3,115 (43.7)	210/492 (42.7)
Without: initiated n/N (%)	243/2,487 (9.8)	49/333 (14.7)
Adjusted OR, fracture diagnosis vs none (95% CI)	6.19 (5.26–7.29)	4.51 (3.09–6.56)

Site-matched fracture diagnosis: S72 for hip and knee operations; S22, S32, M48.4, M48.5 or M80 for spinal operations. Neoplastic/pathological: M84.5, C79.5 or C90. External-cause codes (V, W) were almost never recorded, so trauma mechanism cannot be ascertained. Cells below 5 are masked.

Table S6. Calcium and vitamin D analogue dispensing

	Vertebroplasty/kyphoplasty	Hip fracture internal fixation	Hip arthroplasty (hemi- or total)	Lumbar spinal fusion	Total knee arthroplasty	All operations
No recorded	689	481	720	1,718	811	4,419

initiation within 365 d, n						
Of these, calcium or vitamin D analogue dispensed within 365 d, n (%)	34 (4.9)	29 (6.0)	41 (5.7)	121 (7.0)	36 (4.4)	261 (5.9)
Initiators with calcium or vitamin D analogue, n (%)	120 (19.7)	70 (19.2)	95 (26.8)	96 (26.5)	20 (31.7)	401 (22.9)

Calcium carbonate, calcium citrate, calcitriol and alfacalcidol dispensed at the study hospital within 365 days after the index operation. These agents were not counted as initiation of anti-osteoporosis therapy. Over-the-counter supplements are not captured.

Table S7. Initiation models under alternative treatments of within-patient clustering

Model	Term	Model-based SE (as submitted)	Patient-clustered robust SE (primary analysis)	GEE, exchangeable working correlation	SE ratio, clustered / model-based
Model 1	Vertebroplasty/kyphoplasty	3.15 (2.68–3.72)	3.15 (2.68–3.71)	3.00 (2.58–3.50)	0.982
Model 1	Hip fracture internal fixation	2.61 (2.16–3.16)	2.61 (2.15–3.17)	2.50 (2.08–3.02)	1.021
Model 1	Hip arthroplasty (hemi- or total)	1.84 (1.54–2.20)	1.84 (1.54–2.19)	1.81 (1.53–2.13)	0.978
Model 1	Total knee arthroplasty	0.30 (0.23–0.40)	0.30 (0.23–0.40)	0.32 (0.24–0.41)	0.993
Model 1	Observations / patients / working correlation	6,171	5,739	0.460	
Model 2	Vertebroplasty/kyphoplasty	3.16 (2.68–3.73)	3.16 (2.69–3.72)	2.99 (2.57–3.49)	0.983
Model 2	Hip fracture internal fixation	2.57 (2.13–3.12)	2.57 (2.12–3.13)	2.46 (2.04–2.98)	1.024
Model 2	Hip arthroplasty (hemi- or total)	1.82 (1.52–2.18)	1.82 (1.53–2.17)	1.79 (1.52–2.11)	0.975
Model 2	Total knee arthroplasty	0.28 (0.21–0.37)	0.28 (0.21–0.37)	0.29 (0.23–0.38)	1.002
Model 2	Observations / patients / working correlation	6,171	5,739	0.460	
Model 3	Vertebroplasty/kyphoplasty	3.11 (2.17–4.44)	3.11 (2.18–4.42)	2.66 (1.90–3.72)	0.988
Model 3	Hip fracture internal fixation	2.29 (1.18–4.44)	2.29 (1.15–4.57)	1.61 (0.99–2.60)	1.041
Model 3	Hip arthroplasty (hemi- or total)	1.01 (0.57–1.77)	1.01 (0.57–1.77)	1.02 (0.68–1.54)	1.0
Model 3	Total knee arthroplasty	0.22 (0.10–0.49)	0.22 (0.10–0.49)	0.57 (0.32–1.01)	0.999
Model 3	Observations / patients / working correlation	871	829	0.905	
Model 2, first index operation per patient	Vertebroplasty/kyphoplasty	3.21 (2.70–3.82)			
Model 2, first index operation per patient	Hip fracture internal fixation	2.59 (2.13–3.16)			
Model 2, first index operation per patient	Hip arthroplasty (hemi- or total)	1.91 (1.58–2.30)			
Model 2, first index operation per patient	Total knee arthroplasty	0.28 (0.21–0.38)			
Model 2, first index operation per patient	Observations / events	5,739 / 1,633			
Model 3, first	Vertebroplasty/kyphoplasty	3.04 (2.12–4.38)			

index operation per patient					
Model 3, first index operation per patient	Hip fracture internal fixation	2.61 (1.32–5.18)			
Model 3, first index operation per patient	Hip arthroplasty (hemi- or total)	0.97 (0.54–1.74)			
Model 3, first index operation per patient	Total knee arthroplasty	0.22 (0.10–0.50)			
Model 3, first index operation per patient	Observations / events	829 / 263			

Odds ratios (95% CI) versus lumbar spinal fusion. The primary analysis is logistic regression with a patient-clustered sandwich variance, equivalent to GEE with an independence working correlation. In the rows headed Observations, the three columns give operations, patients and the estimated exchangeable correlation. The first-operation analyses contain one operation per patient, so their observations are independent. The exchangeable fit of Model 3 rests on 42 patients with more than one operation and an estimated correlation of 0.91; because every operation of a patient shares one first-dispensing date, that fit is dominated by within-patient contrasts and is reported for transparency only.

Table S8. Sensitivity analyses of the initiation models

(a) Adding the DXA-to-surgery interval (Model 2: the 2,203 operations with a usable preoperative T-score; Model 3: the 871 with documented osteoporosis)

	**Model 2; without interval**	**Model 2; + interval, per year**	**Model 2; + interval, log(1+years)**	**Model 3; without interval**	**Model 3; + interval, per year**	**Model 3; + interval, log(1+years)**
n	2,203	2,203	2,203	871	871	871
events	439	439	439	274	274	274
Vertebroplasty/kyphoplasty	3.86 (2.95–5.04)	3.86 (2.95–5.05)	3.83 (2.93–5.01)	3.11 (2.18–4.42)	3.13 (2.19–4.47)	3.10 (2.17–4.42)
Hip fracture internal fixation	3.20 (1.89–5.40)	3.47 (2.02–5.94)	3.70 (2.14–6.38)	2.29 (1.15–4.57)	2.65 (1.30–5.38)	2.89 (1.41–5.92)
Hip arthroplasty (hemi- or total)	0.98 (0.64–1.52)	1.04 (0.67–1.62)	1.10 (0.70–1.72)	1.01 (0.57–1.77)	1.08 (0.61–1.92)	1.16 (0.65–2.07)
Total knee arthroplasty	0.23 (0.14–0.41)	0.25 (0.14–0.44)	0.27 (0.15–0.47)	0.22 (0.10–0.49)	0.25 (0.11–0.56)	0.28 (0.13–0.63)
Interval term OR	-	0.94 (0.86–1.03)	0.75 (0.58–0.97)	-	0.89 (0.77–1.03)	0.62 (0.41–0.93)

(b) Restricted to patients aged 50 years or over

**Specification**	**n**	**events**	**Vertebroplasty/kyphoplasty**	**Hip fracture internal fixation**	**Hip arthroplasty (hemi- or total)**	**Total knee arthroplasty**
Model 2 (patient-clustered SE), age ≥ 50 only	6,026	1,746	3.15 (2.68–3.71)	2.54 (2.09–3.09)	1.84 (1.54–2.20)	0.28 (0.21–0.37)

(c) Analyzed operations in patients younger than 50 years

**Operation**	**Analyzed, n**	**Aged <50 y, n (%)**	**Initiated among <50 y, n**
Vertebroplasty/kyphoplasty	1,298	8 (0.6)	<5
Hip fracture internal fixation	845	11 (1.3)	<5
Hip arthroplasty (hemi- or total)	1,074	31 (2.9)	0
Lumbar spinal fusion	2,080	89 (4.3)	<5
Total knee arthroplasty	874	6 (0.7)	0
All	6,171	145 (2.3)	6

(d) Restricted to index operations from 2019 onward (whole look-back coded in ICD-10-CM)

Cohort	n	events	Vertebroplasty/kyphoplasty	Hip fracture internal fixation	Hip arthroplasty (hemi- or total)	Total knee arthroplasty
All analyzed operations	4,132	1,229	2.97 (2.45–3.60)	2.61 (2.06–3.31)	1.71 (1.38–2.12)	0.23 (0.16–0.33)
Documented osteoporosis	679	216	3.45 (2.30–5.17)	2.51 (1.17–5.40)	1.18 (0.61–2.28)	0.13 (0.05–0.38)

(e) Adding cognitive impairment, recurrent oral glucocorticoid dispensing and prior fracture

	All; Model 2	All; + cognitive, glucocorticoid	All; + prior MOF	Documented OP; Model 2	Documented OP; + cognitive, glucocorticoid	Documented OP; + prior MOF
n	6,171	6,171	6,171	871	871	871
events	1,752	1,752	1,752	274	274	274
Vertebroplasty/kyphoplasty	3.16 (2.69–3.72)	3.17 (2.69–3.74)	3.19 (2.71–3.76)	3.11 (2.18–4.42)	3.12 (2.19–4.45)	3.19 (2.24–4.55)
Hip fracture internal fixation	2.57 (2.12–3.13)	2.57 (2.11–3.12)	2.58 (2.12–3.14)	2.29 (1.15–4.57)	2.28 (1.15–4.52)	2.42 (1.17–5.00)
Hip arthroplasty (hemi- or total)	1.82 (1.53–2.17)	1.80 (1.51–2.15)	1.82 (1.52–2.17)	1.01 (0.57–1.77)	0.98 (0.55–1.75)	1.09 (0.60–1.97)
Total knee arthroplasty	0.28 (0.21–0.37)	0.28 (0.21–0.37)	0.28 (0.21–0.38)	0.22 (0.10–0.49)	0.21 (0.10–0.47)	0.22 (0.10–0.48)
Prior major osteoporotic fracture	-	-	0.69 (0.51–0.94)	-	-	0.44 (0.26–0.76)
Dementia/cognitive impairment	-	1.28 (0.98–1.65)	1.29 (0.99–1.67)	-	1.59 (0.81–3.12)	1.80 (0.90–3.63)
Recurrent oral glucocorticoid	-	2.13 (1.64–2.77)	2.10 (1.61–2.73)	-	3.02 (1.59–5.75)	2.87 (1.51–5.45)

Odds ratios (95% CI) versus lumbar spinal fusion from logistic regression with patient-clustered standard errors; all models include the Model 2 covariates.

Table S9. Treatment initiation after lumbar spinal fusion by preoperative densitometry category

Preoperative DXA category	Operations, n	Initiated within 365 days, n (%)	Not initiated, n (%)
Osteoporosis (T <= -2.5)	332	78 (23.5)	254 (76.5)
Osteopenia (-2.5 < T <= -1.0)	526	63 (12.0)	463 (88.0)
Normal (T > -1.0)	232	5 (2.2)	227 (97.8)
Adjusted OR for documented osteoporosis (age- and sex-adjusted, n=1,090)		2.34 (1.61–3.40), p < 0.001	

Categories use the lower of lumbar spine and femoral neck T-score from the last preoperative study.

Table S10. One-year persistence among those who started treatment

Operation	Initiators analyzed, n	Persistent at 1 y, 30-day gap, n (%)	Persistent at 1 y, 60-day gap, n (%)	Short-acting agents only, persistent at 1 y, n (%)	Median days to discontinuation
Vertebroplasty/kyphoplasty	599	394 (65.8)	479 (80.0)	222 (73.0)	280
Hip fracture internal fixation	357	253 (70.9)	293 (82.1)	79 (66.4)	244
Hip arthroplasty (hemi- or total)	347	250 (72.0)	286 (82.4)	87 (70.7)	281
Lumbar spinal fusion	346	207 (59.8)	268 (77.5)	147 (69.7)	280
Total knee arthroplasty	60	44 (73.3)	49 (81.7)	18 (75.0)	373

All operations	1,709	1,148 (67.2)	1,375 (80.5)	553 (70.8)	280
by index agent: Denosumab, 6-monthly	824	672 (81.6)	718 (87.1)		
by index agent: Oral bisphosphonate	203	96 (47.3)	144 (70.9)		
by index agent: Raloxifene, oral	297	128 (43.1)	204 (68.7)		
by index agent: Teriparatide, daily	281	148 (52.7)	205 (73.0)		
by index agent: Zoledronic acid, yearly	104	104 (100.0)	104 (100.0)		

Persistence is continuous coverage from the first prescription with a permissible gap of 30 or 60 days; the 60-day version is the primary. Zoledronic acid is 100% persistent by construction (one yearly infusion covers the whole horizon).

Zoledronic acid re-dosing (yearly agent; persistence above is 100% by construction and is not an adherence result)

	**day 300 to 455**	**day 300 to 545**
Zoledronic acid initiators observable for the window, n	97	90
Second zoledronic acid infusion, n (%; 95% CI)	53 (54.6; 44.7–64.2)	49 (54.4; 44.2–64.3)
Any anti-osteoporosis agent in window, n (%; 95% CI)	58 (59.8; 49.8–69.0)	54 (60.0; 49.7–69.5)

Persistence was analyzed in the 1,709 of 1,752 initiators who had at least 365 days between the first dispensing and the census date. Re-dosing is assessed among zoledronic acid initiators observable for the whole window.

Table S11. Specialty writing the first anti-osteoporosis prescription

**Operation**	**Initiators, n**	**Orthopedics, n (%)**	**Neurosurgery, n (%)**	**Geriatric Hip Fracture Clinic, n (%)**	**Geriatrics, n (%)**	**Hematology /oncology, n (%)**	**Nephrology, n (%)**	**Other departments, n (%)**	**Written as an inpatient, n (%)**	**Median days from surgery to prescription**
Vertebroplasty /kyphoplasty	609	348 (57.1)	244 (40.1)	0 (0.0)	<5 (0.5)	5 (0.8)	<5 (0.7)	5 (0.8)	50 (8.2)	14
Hip fracture internal fixation	364	279 (76.6)	<5 (1.1)	60 (16.5)	15 (4.1)	<5 (0.3)	<5 (0.8)	<5 (0.5)	25 (6.9)	28
Hip arthroplasty (hemi- or total)	354	297 (83.9)	<5 (0.3)	41 (11.6)	8 (2.3)	<5 (0.6)	<5 (0.6)	<5 (0.8)	9 (2.5)	35
Lumbar spinal fusion	362	184 (50.8)	168 (46.4)	0 (0.0)	<5 (0.3)	<5 (1.1)	<5 (0.6)	<5 (0.8)	53 (14.6)	40
Total knee arthroplasty	63	52 (82.5)	5 (7.9)	<5 (1.6)	0 (0.0)	<5 (4.8)	0 (0.0)	<5 (3.2)	<5 (3.2)	123
All operations	1,752	1,160 (66.2)	422 (24.1)	102 (5.8)	27 (1.5)	15 (0.9)	11 (0.6)	15 (0.9)	139 (7.9)	25

Department is taken from the visit attached to the first qualifying prescription. Geriatric Hip Fracture Clinic is a combined geriatric-orthopedic outpatient service.

Table S12. Continuous versus dichotomized T-score: fit and discrimination

Model	Parameters	Partial log-likelihood	AIC	Harrell's C	LRT vs covariates only, p
Covariates only	7	−670.43	1354.9	0.673	-
+ continuous T-score	8	−667.5	1351.0	0.686	0.0155
+ T ≤ −2.5 dichotomy	8	−669.6	1355.2	0.678	0.196

Cox models in 1,971 operations (99 fractures), adjusted for age, sex, comorbidity count and index operation. Difference in Harrell's C, continuous minus dichotomized: 0.008 (bootstrap 95% interval −0.010 to 0.021, patients resampled). The two exposure models are not nested, so no likelihood-ratio test between them is given.

Table S13. Prognostic analysis with alternative blanking periods

	30	90	180	365
Operations, n	2,108	2,058	1,971	1,731
Fractures, n	168	114	99	78
Incidence per 1,000 PY: normal / osteopenia / osteoporosis	8.6 / 22.8 / 35.5	4.1 / 16.6 / 24.8	4.3 / 16.1 / 21.7	3.3 / 14.3 / 20.7
T-score per 1 unit lower, adjusted HR (95% CI)	1.248 (1.082–1.438)	1.309 (1.109–1.545)	1.248 (1.043–1.493)	1.360 (1.109–1.668)
p (continuous)	0.00229	0.00142	0.0154	0.00312
T ≤ −2.5 vs not, adjusted HR (95% CI)	1.484 (1.041–2.115)	1.536 (0.990–2.383)	1.325 (0.827–2.122)	1.566 (0.920–2.664)
p (dichotomy)	0.0291	0.0553	0.242	0.0983

Follow-up for fracture begins the stated number of days after surgery; fractures coded earlier are disregarded. The 180-day analysis is the submitted one. Patient-clustered standard errors, as in Table 4. Absence of recorded treatment is defined over the first 365 days, so only the 365-day analysis is free of conditioning on events after the start of follow-up.

Table S14. Prognostic analysis under alternative censoring rules and with death as a competing event

(a) Hazard ratios under alternative censoring rules (180-day blanking)

	Administrative censoring only (submitted analysis)	Censored at recorded death	Censored at recorded death or last institutional contact
Operations, n	1,971	1,944	1,702
Fractures, n	99	99	99
Person-years	6,446	6,263	4,131
Median follow-up, y	2.3	2.3	1.6
T-score per 1 unit lower, HR (95% CI)	1.248 (1.043–1.493)	1.240 (1.036–1.484)	1.291 (1.070–1.556)
T ≤ −2.5 vs not, HR (95% CI)	1.325 (0.827–2.122)	1.322 (0.826–2.115)	1.430 (0.895–2.284)

(b) Five-year cumulative incidence with and without recorded death as a competing event

	Normal	Osteopenia	Osteoporosis
Operations, n	372	920	679
Fractures, n	6	48	45
Recorded deaths before fracture, n	5	12	7
5-y cumulative incidence, 1 - Kaplan-Meier, % (95% CI)	2.1 (0.9–4.9)	7.0 (5.0–9.7)	11.5 (8.4–15.6)
5-y cumulative incidence, Aalen-Johansen (death competing), % (95% CI)	2.1 (0.8–4.5)	7.0 (4.9–9.5)	11.5 (8.3–15.4)

Fifty-two deaths were recorded among the 1,971 operations, 24 of them before a fracture in (b). Patient-clustered standard errors. Death ascertainment in the institutional data is incomplete, so these analyses bound the effect of recorded deaths only.

Table S15. Adjusted hazard ratios at fixed T-scores from the restricted cubic spline

From the four-knot restricted cubic spline Cox model of Table 4 (n = 1,971, 99 events), adjusted for age, sex, comorbidity count and index operation. Hazard ratios are relative to a T-score of −1.0. See also Figure S4.

T-score	WHO category	Patients with T-score ≤ value, n	Adjusted HR vs T = −1.0 (95% CI)
+0.0	Normal	1,860	0.73 (0.38–1.38)
−1.0	Osteopenia	1,599	1.00 (reference)
−1.5	Osteopenia	1,337	1.28 (1.07–1.51)
−2.0	Osteopenia	996	1.75 (1.24–2.48)
−2.5	Osteoporosis	679	2.25 (1.31–3.86)
−3.0	Osteoporosis	404	2.35 (1.29–4.29)
−3.5	Osteoporosis	204	2.12 (1.16–3.86)
−4.0	Osteoporosis	100	1.76 (0.90–3.44)

Table S16. Comparison of linear, spline, and two-piecewise models for the T-score–fracture relationship

Same cohort and adjustment as Table 4. The spline uses four knots; the two-piecewise (segmented) model places the change in slope at a T-score of −2.0. The p value is the likelihood-ratio test against the linear model; for the two-piecewise model it treats the breakpoint as fixed (1 df) and is therefore anti-conservative. Counting the breakpoint as an estimated parameter (2 df) gives p = 0.055. The breakpoint analysis was post hoc; its stability is examined in Table S17.

Model	df	HR / shape	Non-linearity / threshold p
Linear T-score (per 1 unit lower)	1	1.248 (1.043–1.493)	–
Restricted cubic spline, 4 knots	3	See Figure S4 (ref T = −1.0)	0.052 (χ^2 = 5.90, 2 df)
Two-piecewise, breakpoint T = −2.0	2	Above breakpoint 1.96 / below 0.94 (per unit lower)	0.016 (χ^2 = 5.79, 1 df)

Table S17. Profile likelihood and bootstrap stability of the two-piecewise breakpoint

(a) Profile of the partial likelihood over candidate breakpoints

Candidate breakpoint T	Partial log-likelihood	LR vs linear (chi2)	Fractures at T ≤ K	Fractures at T > K	Operations at T ≤ K
−3.5	−666.07	2.87	15	84	204
−3.4	−665.98	3.04	16	83	231
−3.3	−665.86	3.28	18	81	267
−3.2	−665.77	3.47	22	77	316
−3.1	−665.61	3.79	25	74	359
−3.0	−665.49	4.02	26	73	404
−2.9	−665.23	4.55	28	71	445
−2.8	−664.94	5.12	35	64	503
−2.7	−664.91	5.19	37	62	559
−2.6	−664.78	5.45	43	56	618
−2.5	−664.83	5.34	45	54	679
−2.4	−664.71	5.58	50	49	738
−2.3	−664.71	5.58	53	46	796
−2.2	−664.69	5.62	57	42	850
−2.1	−664.74	5.52	59	40	923
−2.0	−664.61	5.79	70	29	996
−1.9	−664.99	5.03	74	25	1,071
−1.8	−665.33	4.35	76	23	1,151
−1.7	−665.54	3.92	80	19	1,214
−1.6	−665.84	3.33	82	17	1,284
−1.5	−666.11	2.78	85	14	1,337
−1.4	−666.41	2.18	87	12	1,395
−1.3	−666.68	1.65	90	9	1,450
−1.2	−666.94	1.12	92	7	1,504
−1.1	−667.17	0.66	93	6	1,555
−1.0	−667.34	0.33	93	6	1,599

(b) Selected breakpoint and its uncertainty

Item	Value
Knots (4-knot RCS), T-score	−4.00, −2.40, −1.60, 0.10
Candidate range	−3.5 to −1.0 in steps of 0.1 (26 values)
Selected breakpoint	−2.0
Profile-likelihood 95% interval	−3.5 to −1.4
Fractures / operations at T ≤ breakpoint	70 / 996
Fractures / operations at T > breakpoint	29 / 975
LRT two-piecewise vs linear, 1 df (breakpoint treated as fixed)	$\chi^2 = 5.79$, p = 0.016
LRT two-piecewise vs linear, 2 df (breakpoint counted as a parameter)	$\chi^2 = 5.79$, p = 0.055
Bootstrap resamples (patients resampled, breakpoint re-selected each time)	500
Bootstrap breakpoint, median (2.5th-97.5th percentile)	−2.2 (−3.5 to −1.0)
Resamples selecting a breakpoint within ±0.3 of the original, %	48.8
Resamples selecting a breakpoint above −2.5 (in the osteopenic range), %	61.6
HR per unit lower above breakpoint: original; bootstrap 95% percentile interval	1.96; 0.87–5.03
HR per unit lower below breakpoint: original; bootstrap 95% percentile interval	0.94; 0.28–1.38

(c) Breakpoint selected in each bootstrap resample

Breakpoint	Resamples
−3.5	37.0
−3.4	3.0
−3.3	6.0
−3.2	7.0
−3.1	7.0
−3.0	1.0
−2.9	4.0

−2.8	69.0
−2.7	6.0
−2.6	50.0
−2.5	2.0
−2.4	30.0
−2.3	7.0
−2.2	22.0
−2.1	5.0
−2.0	189.0
−1.9	9.0
−1.8	1.0
−1.7	11.0
−1.6	3.0
−1.5	6.0
−1.4	2.0
−1.3	2.0
−1.2	1.0
−1.0	20.0

Two-piecewise Cox model with the Table 4 covariates. LR: likelihood-ratio statistic against the single-slope model. The profile interval and the bootstrap distribution both reach the limits of the candidate grid (−3.5 and −1.0). The breakpoint analysis was post hoc. The profile is nearly flat between −2.9 and −2.0 and the bootstrap distribution is wide, so the location of any change in slope is not identifiable.

Table S18. Treated versus untreated: why these data cannot support a causal estimate

Analysis	Operations, n	Fractures, n	Hazard ratio for treatment (95% CI)	p value	Comment
Primary: IPTW including preoperative T-score	1,623	70	1.74 (0.95-3.18)	0.0711	E-value 2.88 (point), 1.27 (CI limit) - not usable because weighting did not achieve balance
Same cohort, T-score removed from the propensity model	1,623		1.85 (1.03-3.30)	0.0387	Adding T-score barely moves the estimate: the imbalance is too large to be fixed by weighting
Larger cohort in which no T-score exists at all	4,872	375	1.18 (0.93-1.51)	0.173	A database without bone density cannot detect the imbalance that invalidates this comparison
Complication: Mechanical implant complication	1,623	0 treated / 16 untreated	Not estimable		complete separation: no events in one arm
Complication: Periprosthetic fracture	1,623	0 treated / 4 untreated	Not estimable		complete separation: no events in one arm
Complication: Implant infection [negative	1,623	1 treated / 8 untreated	Not estimable		fewer than 10 events in total

control]					
Pre-specified verdict					Supplementary only: weighting did not achieve balance, so no causal reading is available whatever the estimate shows

Pre-specified before the analysis was run: an estimate would enter the main results only if the hazard ratio favoured treatment, the E-value reached 2, weighting achieved balance (all |SMD| < 0.1), and a negative-control outcome showed no association. Balance was not achieved, so the pre-specified rule places the whole analysis here regardless of what the estimate shows.

Table S19. Covariate balance before and after inverse-probability weighting

**Covariate**	**SMD before weighting**	**SMD after weighting**	**Balanced after weighting (|SMD| < 0.1)**
Age at surgery	0.438	0.165	NO
Female sex	0.061	0.063	yes
Preoperative T-score	-0.697	-0.313	NO
Comorbidity count	0.348	0.051	yes
Outpatient visits (log)	0.118	-0.026	yes
Admissions in prior 3 y	0.013	0.017	yes

Standardized mean differences. Comorbidity burden and healthcare utilization balance well; preoperative T-score and age do not. This is the diagnostic that determined the verdict in Table S18.

Supplementary Figures

Figure S1

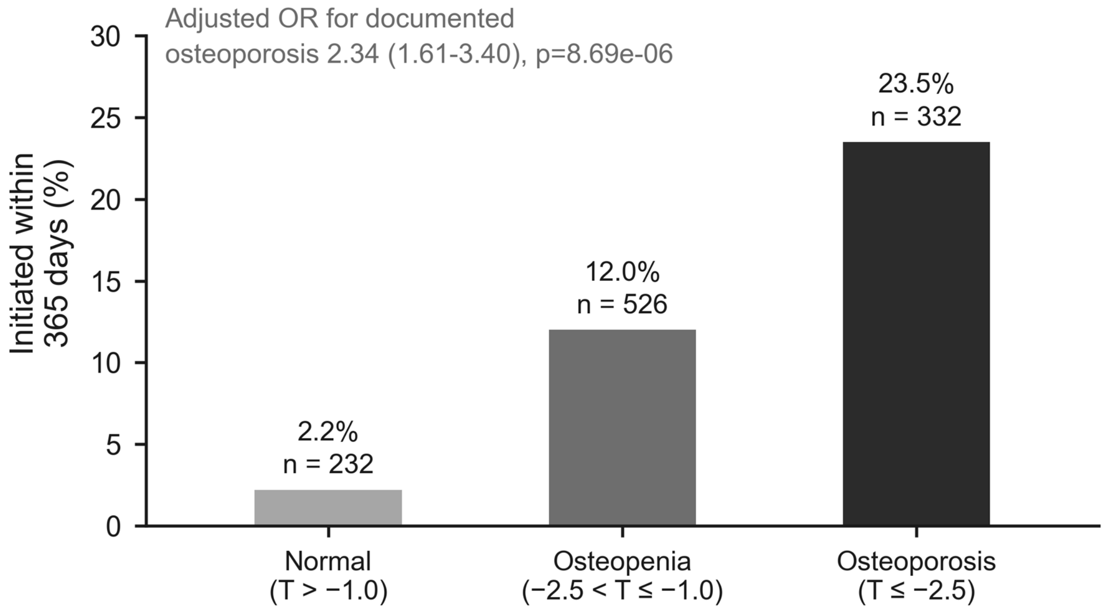

Figure S1. Initiation after lumbar fusion by preoperative densitometry category. Categories use the lower of lumbar spine and femoral neck T-score from the last preoperative study.

Figure S2

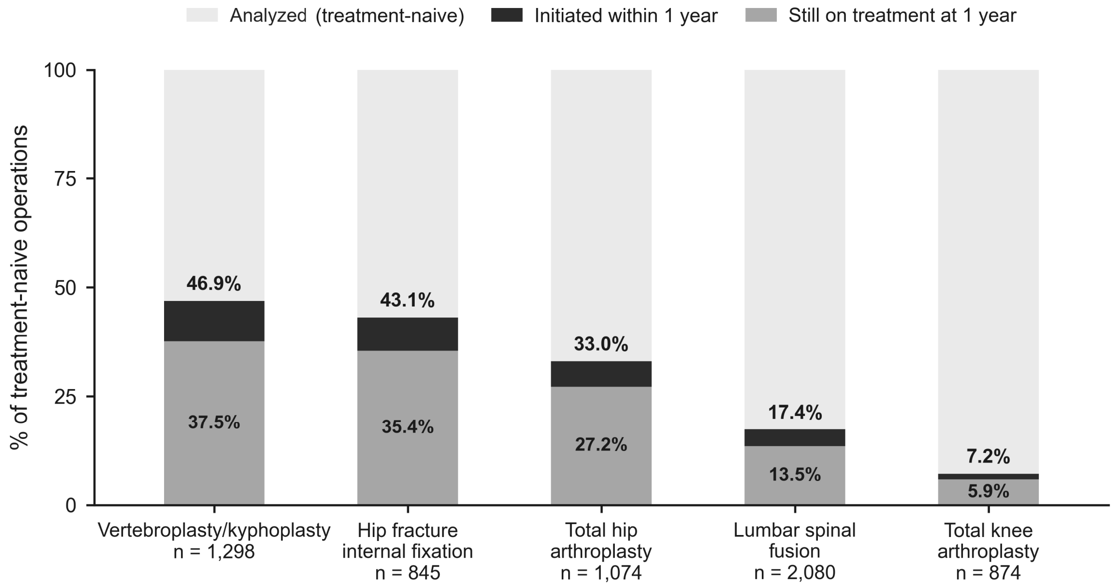

Figure S2. The osteoporosis care cascade after orthopedic surgery. For each operation: all treatment-naïve operations (grey), those initiating within one year (blue), and those still on treatment at one year (green). Almost the entire loss occurs at the first step.

Figure S3

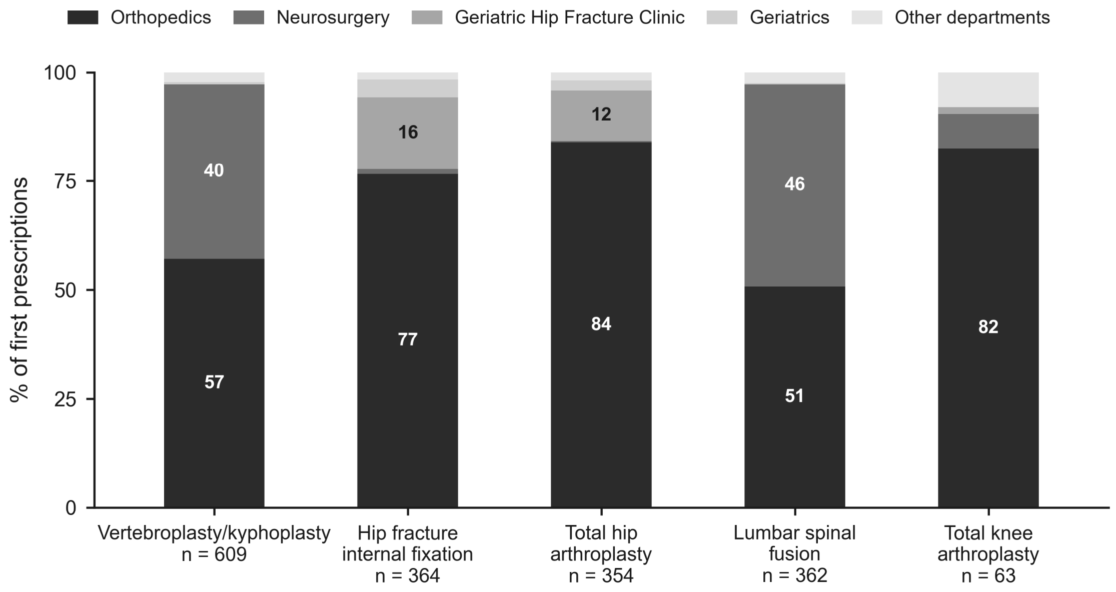

Figure S3. Which specialty writes the first anti-osteoporosis prescription. Departments contributing at least 1% are shown separately; the remainder are pooled.

Figure S4

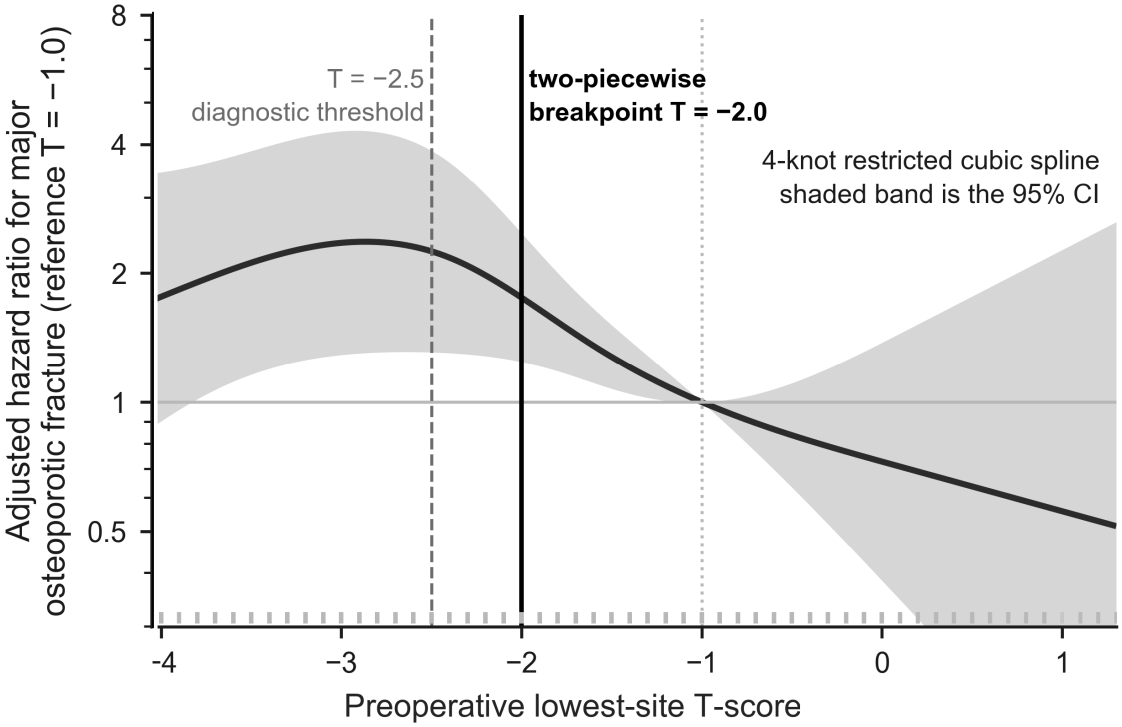

Figure S4. Dose-response of preoperative T-score and major osteoporotic fracture. Adjusted hazard ratio for major osteoporotic fracture (solid line) with 95% confidence interval (shaded band), from a four-knot restricted cubic spline Cox model of the continuous preoperative lowest-site T-score (reference T = −1.0), adjusted for age, sex, comorbidity count and index operation; same cohort as Table 4 (n = 1,971, 99 events). The dashed vertical line marks the −2.5 diagnostic threshold. The solid vertical line marks the post hoc two-piecewise breakpoint at T = −2.0; its location is not identifiable from these data (Table S17) and it should not be read as a threshold. Departure from log-linearity was of borderline significance (p = 0.052). Incomplete death ascertainment is expected to bias the curve toward the null.
